# Truss sizing optimum design using a metaheuristic approach: Connected banking system

**DOI:** 10.1016/j.heliyon.2024.e39308

**Published:** 2024-10-16

**Authors:** Mehrdad Nemati, Yousef Zandi, Jamshid Sabouri

**Affiliations:** Department of Civil Engineering, Tabriz Branch, Islamic Azad University, Tabriz, Iran

**Keywords:** Metaheuristics, Optimum design, Truss structure, Optimization, Algorithm design

## Abstract

Several methods have been used to solve structural optimum design problems since the creation of a need for light weight design of structures and there is still no single method for solving the optimum design problems in structural engineering field that is capable of providing efficient solutions to all of the structural optimum design problems. Therefore, there are several proposed and utilized methods to deal with optimum design issues and problems, that sometimes give promising results and sometimes the solutions are quite unacceptable. This issue with metaheuristic algorithms, which are suitable approaches to solve these set of problems, is quite usual and is supported by the “No Free Lunch theorem”. Researchers try harder than the past to propose methods capable of presenting robust and optimal solutions in a wider range of structural optimum design problems, so that to find an algorithm that can cover a wider range of structural optimization problems and obtain a better optimum design. Truss structures are one of these problems which have extremely complex search spaces to conduct search procedures by metaheuristic algorithms. This paper proposes a method for optimum design of truss sizing problems. The presented method is used against 6 well-known benchmark truss structures (10 bar, 17 bar, 18 bar, 25 bar, 72 bar and 120 bar) and its results are compared with some of the available studies in the literature. The performance of the presented algorithm can be considered as very acceptable.

## Highlights

• Truss sizing optimum design problems are addressed with a new approach.
• The CBSO algorithm was utilized to solve truss optimum design problems.
• The performance of the CBSO in dealing with truss optimum design problems is investigated and analyzed.
• Promising optimum design results are achieved using the CBSO algorithm.

## 1 Introduction

Substantial progress in recent years has been made in optimum design of truss structures using metaheuristic algorithms, which creates a competitive research field among the active researchers in the literature. Developments in metaheuristic algorithms and advancements in engineering design problems have led the optimum design to an extensive area, where new problems arise every day and these problems can be tackled by modified, upgraded, hybridized and novel optimization algorithms. Truss optimum design problems which are considered as a branch of engineering design problems, can be classified into three major classes: shape design, topology design and size design. The last category deals with choosing cross-sections of truss members which do not violate the defined constraints of optimum design problem, and is about finding an optimum design for truss structure with a minimized weight. Achieving this goal can be quite complex and may need a great amount of time to assess and evaluate different structural designs to find an optimum solution among them, since there is no information on optimum sections at the beginning of solving these structural optimization problems.

Methods capable of being computationally efficient in engineering design problems are significantly required and needed in this field, because truss structures categorized in large scale structures, necessitate utilizing efficient algorithms for their designs and analyses [1]. Based on the judgment that a design candidate can be worthy for being investigated by a novel method which uses a machine learning algorithm, namely k-nearest neighbor comparison combined with Rao optimization method, a new approach for solving discrete size optimization of truss structures was developed [2]. An optimum design of truss bridges subjected to excessive temperature changes, was presented by Keleş et al., using cuckoo search algorithm [3]. In a study conducted by Khodadadi et al., eight well-known metaheuristic algorithms were used to find an optimum design for shape and size optimization of three truss structures with stress and displacement constraints [4]. Shape and size optimum design of truss structures with dynamic constraints using a novel algorithm with a combined greedy search method was studied by Etaati et al. [5]. Sizing optimization of five famous truss structures with fixed-geometry having discrete and continuous decision variables under stress and displacement constraints subjected to multiple loading cases using Bonobo optimizer algorithm was investigated in Ref. [6].

Two different tower structures were studied using the parameter-less population pyramid by Gandomi and Goldman [7]. Sizing, layout and topology optimum design of truss structures were evaluated using a non-parametric optimization algorithm called Jaya [8]. Kaveh et al., used a shuffling approach along with a local minima stagnation prevention method to enhance the performance of Jaya algorithm, which is known as improved shuffled based Jaya algorithm to investigate the efficiency of their proposed method in solving structural optimization problems with discrete decision variables [9]. A novel metaheuristic optimization algorithm was developed by Kaveh and Dadras Eslamlou, and then a chaotic version of it was utilized to solve optimum design problems with multiple frequency constraints [10]. An improved version of the arithmetic optimization algorithm for optimum design of skeletal structures was proposed in order to have a better exploration and exploitation balance and capability, in comparison to the original version of the arithmetic optimization algorithm, and to reduce the need to configure specific parameters of the original algorithm [11]. Analyzing different applications of metaheuristics in solving optimum design of truss structures was discussed by Renkavieski and Parpinelli. Their research indicated that optimum design of trusses is an active and challenging research field [12]. Adopting a novel deep neural network-based surrogate model for optimum design of truss structures having geometrically nonlinear behavior, in which the displacements of the analyzed trusses were predicted using the ability of the deep neural network model to learn the relationship between the inputs and the outputs was carried out by Mai et al. [13]. Considering this fact that truss optimization is a challenging optimization problem and requires stochastic methods to be solved, it can be stated that improving the optimization method in terms of being effective and efficient is imperative in dealing with truss optimization problems. Therefore, integrating genetic algorithm and deep neural network during the optimization process, for creating an optimization prediction procedure that was analyzed by Liu and Xia, was quite effective and robust [14]. Improving the computational efficiency in structural optimum design problems was studied by Cao et al., and a reduction in the number of structural analysis by metaheuristic algorithms, approximately about 80 percent, was achieved by their proposed method [15]. Taking advantage of Rao algorithms and proposing a modified Rao algorithm hybridized with the feasible boundary search method that maintains the parameter-freeness and simplicity of Rao algorithms, was investigated in solving optimal truss sizing problems by Pham and Tran [16]. Öztürk and Kahraman, conducted a study on the optimum design of truss structures to investigate existing issues in the optimization of truss bar problems. Computational complexity, utilizing different experimental settings of various algorithms and analyzing algorithms that have great success rate in dealing with truss optimization problems, were assessed in their study [17]. Electromagnetism-like Firefly algorithm was developed to solve discrete structural optimization problems and proved that it can provide great solution accuracy and convergence speed when dealing with truss optimization problems [18]. Enhancing the performance of a swarm intelligence-based algorithm inspired by the herding behavior of shepherds in nature, and utilizing it as an optimizer for optimum design of large-scale space structures was evaluated by Kaveh et al. [19]. Examining the performance of SHADE algorithm in size optimization of truss optimum design problems with multiple frequency constraints was carried out by Kaveh et al., and indicated that the obtained numerical results in their study were more effective and superior than the other compared methods and solving the truss optimization problems required fewer number of structural analysis than the compared methods [20].

Metaheuristics are employed widely in optimum design of truss structures and can be considered quite efficient in searching large complex space of truss optimization problems. However, due to the “No Free Lunch” theorem, these methods do not always have the capability of solving every existing optimization problem efficiently. Therefore, novel, hybridized and upgraded metaheuristics, are presented to overcome this issue. Some metaheuristic algorithms can provide robust results in solving truss structures optimum design problems, while others may present weak results. Genetic algorithm has been successfully used for structural optimum design problems by many researchers, such as a research conducted by Cheng, to optimize steel truss arch bridges where a hybrid genetic algorithm was developed and utilized for solving a truss bridge with a span of 552 m [21]. Optimum design of skeletal structures including truss structures, was analyzed using a metaheuristic algorithm called imperialist competitive algorithm [22]. Optimum truss structures design subjected to static and dynamic constraints such as maximum allowable stress, maximum allowable deflection, critical buckling load and natural frequency constraints with continuous and discrete decision variables was investigated using a hybrid genetic algorithm, and the efficiency of the presented method over other existing methods in the literature was observed for most of the problems that were analyzed [23]. Hybrid harmony search JAYA algorithm was used by Degertekin et al., in order to optimize large scale truss structures and their results indicated that the hybridization of harmony search and JAYA algorithms was quite successful and efficient in the optimum design of three large scale truss structures with nonlinear constraints [24]. Hybridizing two metaheuristic algorithms, one inspired by nature and the other inspired by swarm behavior, was the intention of a study for optimizing a 72-bar truss structure to find its minimum weight [25]. An amendment of Dragonfly optimization algorithm was utilized in optimum design of steel truss structures with discrete decision variables and the effectiveness and improvements of the modified version of the Dragonfly algorithm was revealed in the research of Jawad et al. [26]. A parameter-free version of Jaya algorithm was proposed by Degertekin et al., for weight minimization of size and layout optimum design of truss structures having a range of decision variables from 8 to 59. The performance of the proposed method was considerably robust, since it could outperform some of the state-of-the-art optimization algorithms in terms of optimum truss weight design and fast convergence towards optimum solution [27]. In order to enhance the performance of discrete truss size optimum design using metaheuristic algorithms, modified versions of dragonfly, teaching-learning-based, whale, heat transfer search and ant lion optimizer algorithms were presented and used in solving the truss optimization problems [28].

Being inspired by a trinary phase model of important aspects of hippopotamus behaviors such as specific mechanisms for avoiding predators, being protected from them, the hippopotamus optimizer(HO) algorithm was utilized to optimize five truss structures. Fast convergence of the HO and efficient solutions provided by it indicated that the utilized method was robust and strong [29]. Multi objective version of cheetah optimizer(MOCO) algorithm was used to address optimum design of truss structures. The MOCO is based on some hunting strategies that cheetahs use in nature for finding and attacking preys such as waiting for the attack and conducting attack and returning to their region. An outstanding approach was observed in the achieved results by the MOCO which shows the great ability of the developed algorithm in dealing with truss structural optimum design problems [30]. In order to investigate the performance of many objective truss optimization problems which are rarely addressed in the literature, eighteen optimization algorithms were utilized to analyze their performance in dealing with truss optimum design problems [31]. An improved version of teaching-learning based optimization algorithm incorporating multiple teachers approach was used as an optimizer method to solve topology, layout and size optimization of space and planar truss problems. Great and robust performance of the utilized method was observed and achieved by the developed method [32]. Solving multiple conflicting objectives in optimization problems such as truss structures optimum design, can be quite complex and challenging. A multi-objective version of multiverse optimizer algorithm was utilized to solve optimum design problems particularly truss optimum design problems. The obtained results showed very promising performance of the utilized method [33]. Inspired by the imitating behavior of sheep within the flock, an improved version of follow the leader algorithm was developed, and in order to evaluate its efficiency, it was tested on some of the well-known truss optimum design problems. The developed method achieved promising results that could compete with the state-of-the-art algorithms [34]. There are also important number of studies incorporated metaheuristics to solve different important optimization problems which present interesting and promising results [[35], [36], [37], [38], [39], [40], [41], [42], [43], [44], [45], [46], [47], [48], [49], [50], [51], [52], [53], [54], [55], [56], [57], [58], [59], [60], [61]].

The main contribution of this work is to present, develop and use a metaheuristic algorithm for utilizing in truss sizing optimization problems which is a parameter-free optimization algorithm, and to compare its results with the results of previous studies. The developed algorithm is comprised of three phases for searching search space of optimization problems. It is inspired by financial transactions between different banks, which uses different strategies for solving optimization problems efficiently and can provide very acceptable results in terms of quality of the optimum solutions. The performance of the presented optimization method is tested against six truss structures to find a minimum weight of these structures having different constraints and various numbers of members connected to each other such as 10, 17, 18, 25, 72 and 120 bars. The motivation behind the design of a metaheuristic presented in this paper was to develop a metaheuristic algorithm with no extra parameters needed to be configured and also providing a capability of fast convergence in solving truss optimization problems.

## 2 Problem formulation

Sizing optimization of truss structures, aims to minimize truss weight in a way that optimal values for area of cross sections be determined by an optimization algorithm through iterations and tweaks of the cross sections. This task contains some constraints which make the work harder and therefore, size optimization problems in structural engineering can be quite challenging.

### 2.1 Optimization problem formulation

The size optimization problem for trusses can be mathematically expressed as follows:

(1) $$Find:X=\left[x_{1},x_{2},\ldots ,x_{a},x_{Nv}\right],x^{\min}\leq x_{a}\leq x^{\max}$$

(2) $$To\;minimize:W=\sum_{a=1}^{Nv}x_{a}\sum_{b=1}^{{Nm}_{a}}\rho_{b^{L}b}$$

Where the *X* is comprised of design variables, *Nv* is the total number of design variables *x*_*a*_ is the design variable *a*, *x*^*min*^ and *x*^*max*^ are the minimum and the maximum possible values for the design variables, respectively. *W,* denotes the weight of truss structure, $\rho_{b}$ and $L_{b}$ are the density and length of truss members, respectively. ${Nm}_{a}$, shows the number of members existing in group *a*.

### 2.2 Handling constraints of optimization problem

Since metaheuristics are usually designed for solving unconstrained optimization problems, and truss optimum design problems have some constraints such as allowable displacement, allowable stress and buckling limits, therefore the truss optimization problem should be converted to an unconstrained optimization problem using a constraint handling methodology. The constraints should be formulated in a way that can convert the constrained optimization problem to an unconstrained optimization problem. This can be done by defining the constraints mathematically as follows:

(3) $$g_{i}^{\sigma }\left(x\right)=\frac{\left|\sigma_{i}\left(x\right)\right|-\sigma_{\max}}{\sigma_{\max}}\leq 0\;i=1,2,3,\ldots ,nE$$

(4) $$g_{j}^{\delta }\left(x\right)=\frac{|\delta_{j}\left(x\right)|-\delta_{\max}}{\delta_{\max}}\leq 0\;j=1,2,3,\ldots ,nN$$

(5) $$g_{k}^{b}\left(x\right)=\frac{|\sigma_{k}^{b}\left(x\right)|-\sigma_{\max}^{b}}{\sigma_{\max}^{b}}\leq 0\;k=1,2,3,\ldots ,nC$$

$g_{i}^{\sigma }\left(x\right)$, $\sigma_{\max}$, $g_{j}^{\delta }\left(x\right)$, $\delta_{\max}$, $g_{k}^{b}\left(x\right)$, $\sigma_{\max}^{b}$ indicate the following constraints,respectively: normalized normal stress value of the *ith* member, the maximum allowable normal stress limit for tension and compression, the normalized displacement value of the *jth* node, the maximum allowable nodal displacement value, the normalized buckling value of the *kth* member in compression, the critical buckling stress value when a compression member fails. In equations (3), (4), (5), the number of structural elements, the number of nodes in truss and the number of structural members in compression are shown by *nE, nN* and *nC*, respectively. There are some governing rules for the constraint values presented above which are as follows:

If the value of the constraints $g_{i}\left(x\right)$, $g_{j}\left(x\right)$ or $g_{k}\left(x\right)$ are greater than zero, then the constraints will have a value equal to $g_{i}\left(x\right),g_{j}\left(x\right)\;\text{or}\;g_{k}\left(x\right)$, respectively; otherwise the constraints will have a value of zero. In a truss design optimization problem, if there were some violations of constraints, the total constraint violation will be computed so that all of the normalized values of stresses and displacements will be summed and the penalty values will be presented for all of the truss members and nodes, as a single value as follows:

(6) $$C=\sum_{i=1}^{m}C_{i}=\sum_{i=1}^{nE}\left(\frac{\left|\sigma_{i}\left(x\right)\right|-\sigma_{\max}}{\sigma_{\max}}\right)+\sum_{j=1}^{nN}\left(\frac{\left|\delta_{j}\left(x\right)\right|-\delta_{\max}}{\delta_{\max}}\right)+\sum_{k=1}^{nC}\left(\frac{\left|\sigma_{k}^{b}\left(x\right)\right|-\sigma_{\max}^{b}}{\sigma_{\max}^{b}}\right)$$

where *m* indicates the total number of the defined constraints for truss structure optimum design problem. The fitness function that is to be minimized in this paper takes the following form:

(7) $$\emptyset \left(X\right)=\left(\sum_{a=1}^{Nv}x_{a}\sum_{b=1}^{{Nm}_{a}}\rho_{b^{L}b}\;\right)*{\left(1+C\right)}^{2}$$

## 3 Connected Banking System Optimization algorithm

### 3.1 Inspiration

There are different ways of connecting banks to each other in a system for doing transactions and submitting them. These different ways and methods can be considered as approaches to solve complex optimization problems such as sizing truss structural optimum design problems. Each bank can decide to choose a route for its money transferring and any route which does a transaction faster than the other methods, can be considered as an optimum solution for an optimization problem after a maximum number of iterations of the proposed algorithm. Also, each bank may face financial problems sometimes that can affect other banks connected to the financial system, and cause to crash the system which will be considered in the presented metaheuristic algorithm, and for conducting a safe transaction, it is assumed to have a high value for the crash probability to amend and reconsider the route by the algorithm so that the transaction can be verified. The global financial system that is highly interconnected, and causes banks to interact with each other both directly and indirectly. Since these banks are highly interconnected, issues that may rise in one section of the system can be easily transmitted to other sections of this financial system. Financial crisis in 2008 affected banking systems due to the highly interconnected nature of the global financial system. Therefore, difficulties happened in many banks across these financial systems. This can result in real problems in economy and affect the economic systems of countries connected to the system. The banking system, can be connected directly using bilateral transactions and if the probability of successfully connecting between two banks was high, it could also affect the other banks in the system. There are also some banks which are called core banks and can dominate the banks existing in the system and any problem that could happen to the core banks would absolutely affect all of the other banks and the problem might propagate in the whole system. It is possible that sometimes a bank in the system can be helpful to the other banks and to become a core bank that will dominate the other banks and the whole system. Based on this interconnectedness of the financial banking system, a metaheuristic algorithm is proposed here, which is designed to optimize 6 well-known truss structures. The proposed method is called Connected Banking System Optimization(CBSO) algorithm. The banking system starts with some random routes connecting the banks existing in the system, which submit different transactions among the connected banks. Each route is considered as a search agent and the amount of time that takes a transaction to successfully occur, is the quality of objective function, which highly relies on the route connecting the banks in the system. The whole algorithm is comprised of three phases and an updating mechanism for each phase, so that the routes between banks can be encrypted and since the probability of happening a crash in the system is relatively high, that is assumed to be twenty percent in each phase of the algorithm, the routes may change, and a completely new solution can be produced based on an already existing solution in the current iteration of the presented algorithm. In the first phase, the original transaction sender bank, creates a route among the banks to the transaction receiver bank upon the information it already has about the so far best possible and available route towards the destination bank in the system. In the second phase of the algorithm, the best route will continue submitting half of the transactions and a random route between the available routes will be selected to help sending and receiving the transactions. The other half of the transactions will be submitted according to another random route and again the so far best available route for submitting the transactions, but the original bank decides where to send them and chooses the route of transferring money between banks. At the final phase of the algorithm, the best route which has an overall information of the available routes, decides to pick two random routes and by collaborating with the two random routes, it chooses the routes that will have a higher speed of doing transactions and take less time than the other available routes, leading the proposed algorithm to the best solution of optimization problem. It is also important to consider the probable crash in the system and change the current selected route to another one which hopefully will not result in another crash in the system. If there was no crash in the system, then the information of the routes will be encrypted so that only the sender and the receiver will be able to read and understand the transaction information.

### 3.2 Mathematical model

A mathematical model is presented here for the proposed CBSO algorithm, that shows the formulated concepts of the presented metaheuristic method. The first stage of the presented method, determines the initial positions of the search agents of the algorithm in search space of optimization problem. Each search agent, is denoted by X_i_, which are also called candidate solutions. It can be mathematically formulated as follows:

(8) $$X=\left[\begin{array}{c} X_{1}\\ X_{2}\\ \vdots \\ X_{i}\\ \vdots \\ X_{n} \end{array}\right]=\left[\begin{array}{cccccc} x_{1}^{1} & x_{1}^{2} & \ldots & x_{1}^{j} & \ldots & x_{1}^{d}\\ x_{2}^{1} & x_{2}^{2} & \ldots & x_{2}^{j} & \ldots & x_{2}^{d}\\ \vdots & \vdots & \vdots & \vdots & \ddots & \vdots \\ x_{i}^{1} & x_{i}^{2} & \ldots & x_{i}^{j} & \ldots & x_{i}^{d}\\ \vdots & \vdots & \vdots & \vdots & \ddots & \vdots \\ x_{n}^{1} & x_{n}^{2} & \ldots & x_{n}^{j} & \ldots & x_{n}^{d} \end{array}\right],\left\{ \;\begin{array}{c} i=1,2,\ldots ,n\text{.}\\ j=1,2,\ldots ,d\text{.} \end{array}\right.$$

(9) $$x_{i}^{j}=x_{i,\min}^{j}+rand.\left(x_{i,\max}^{j}-x_{i,\min}^{j}\right),\left\{ \;\begin{array}{c} \;i=1,2,\ldots ,n\text{.}\\ \;j=1,2,\ldots ,d\text{.} \end{array}\right.$$

Where n, d, $x_{i}^{j}$, $x_{i,\max}^{j}$, $x_{i,\min}^{j}$, and *rand* represent the number of search agents(candidate solutions), the dimension of optimization problem, the *jth* variable in the *ith* initial position, upper bound of the *ith* search agent of the *jth* decision variable, lower bound of the *ith* search agent of the *jth* decision variable and a random number in the interval of 0 and 1, respectively. equations (8), (9) are utilized to create the initial search agents in search space of optimization problem and contain the status of banks which are connected to each other. Next stages of the algorithm procedure show the changes in the status of the banks and form the overall behavior of the financial banking system, by utilizing some strategies.

Each iteration of the CBSO algorithm, tries to update the status of banking system and each route connecting banks to each other, in order to evaluate the new values of objective function of optimization problem that is being solved. The proposed algorithm utilizes three phases for finding optimum solutions and the phases can be mathematically formulated as follows:

Phase1: :

If the progress in the search process of the CBSO algorithm is less than 20 percent of the maximum number of iterations, then the updated positions should be tweaked using the following equation:

(10) $$\vec{{NewX}_{i}}=\vec{X_{i}+}\vec{{\text{RN}}_{1}}⊗\left(\left(R1.\vec{BB}-\vec{X_{i}}\right)\right)\;i=1,\ldots n$$

where X_i_, NewX_i_, RN_1_, R1 and BB denote the search agents at iteration *i*, new generation of the algorithm's search agents, a vector of random numbers with normal distribution, a random number having a value in the interval of zero and one, and the best solution so far found.

Phase2: :The next phase of the CBSO algorithm begins when the search progress of the algorithm reaches 20 percent of the maximum number of iterations and it continues until it is less than or equal to 40 percent of the maximum number of iterations. At this phase, the search agents are split in two halves and each half is updated using a different formula as follows:

(11) $$\left\{ \begin{array}{c} \vec{{NewX}_{i}}=\vec{BB}+rand.\left(1-\frac{t}{t_{\max}}\right).\left(\vec{{\text{RN}}_{2}}⊗\left(R2.\vec{BB}-\vec{{\text{XS}1}_{i}}\right)\right)\\ \vec{{NewX}_{i}}=\vec{X_{i}}+\vec{LD}⊗\left(\vec{BB}-\vec{LD}⊗\vec{{\text{XS}2}_{i}}\right) \end{array}\right.i=1,\ldots n$$

Where the $\vec{\;{NewX}_{i}}$ value assignment is determined using two different ways, in a manner that if the search agents are placed in the first half, the algorithm will assign the $\vec{\;{NewX}_{i}}$ value using the first equation provided in the expression (11) and otherwise the second equation of expression (11) will determine the $\vec{\;{NewX}_{i}}$ values. $\vec{\;LD}$ denotes a vector of random variables with Lévy distribution. In equation (11), *t* and *t*_max_ show the current iteration value and the maximum number of iterations value, respectively. $\vec{\;{\text{XS}1}_{i}}$ and $\vec{\;{\text{XS}2}_{i}}$ indicate two random search agents chosen from the available population of search agents. RN_2_, R2 show a vector of random numbers with normal distributions, and a random number having a value in the interval of [0,1], respectively.

The final phase of the CBSO algorithm attempts to take the advantage of the power of exploitation which is used for local searching of promising areas found during the search process of the proposed optimization algorithm.

Phase3: :

(12) $$\vec{{NewX}_{i}}=\vec{BB}+rand.\left(1-\frac{t}{t_{\max}}\right).\left(\vec{LD}⊗\left(\vec{LD}⊗\vec{{\text{XS}1}_{i}}-\vec{{\text{XS}2}_{i}}\right)\right)\;i=1,\ldots n$$

For enhancing the performance of the presented method, a robust search mechanism is considered along with the search phases that were defined above, and updates the solutions at each iteration, according to the following:

Since there are possibilities of disruptions in the banking systems existing in the route between two banks, a malfunctioning banking system between the two banks and along the route which connects the two banks, can cause the system to crash. Therefore, this possibility must be taken into account and the route from the transaction sender bank to the transaction receiver bank must be changed in an efficient manner. The possibility of occurring a crash in the banking system is considered twenty percent and also for considering security threats such as various cyber-attacks, all of the transactions are encoded, and this security measure can also affect the route change mechanism of the presented algorithm in a way that predicting the transaction route becomes really hard, and the possibility of detecting it by cyber criminals, decreases. Therefore, if a random number between zero and one, is in the range of 0 and 0.2, then there is a crash and a need for the route change is imperative. Otherwise, the encoding system is activated and the route which will determine the transaction target bank changes accordingly. The crash and encoding mechanisms can be mathematically formulated as follows:

(13) $$\left\{ \begin{array}{c} \vec{{UPX}_{i}}=\vec{{NewX}_{i}}+rand.\left(rand.\left(1-\frac{t}{t_{\max}}\right).LB+rand.\left(UB-LB\right)\right)\;if\;RU<0.2\\ \vec{{UPX}_{i}}=\vec{{NewX}_{i}}+\left(rand.rand\right).\left(\vec{{\text{XS}1}_{i}}-\vec{{\text{XS}2}_{i}}\right)\;Otherwsie\text{.} \end{array}\right.$$

Where $\vec{{UPX}_{i}}$ is the updated answer considering the crash and encoding mechanisms, *rand* is a random number in the interval of zero and one, LB and UB are the lower bounds and upper bounds of the optimization problem, respectively. *RU* is a random number in the interval of zero and one, that determines the possibilities of crash and activation of the encoding system.

## 4 Numerical examples

For performance assessment of the proposed optimization algorithm, a total number of six truss structures with different constraints are considered as benchmarks, and the effectiveness of the CBSO algorithm is analyzed and evaluated. The considered truss structures have member sizes of 10 bar, 17 bar, 18 bar, 25 bar, 72 bar and 120 bar. The structure of all problems is analyzed using direct stiffness method and optimized utilizing the CBSO algorithm. The results of the optimum design are compared and validated with existing published results in the literature. In order to evaluate statistical performance of the algorithm and acquire a better understanding of the algorithm's performance, each problem was solved 30 times using the CBSO algorithm and the best, mean weight and the standard deviation values of the 30 independent runs are presented. The CBSO algorithm and the truss problems are coded in the MATLAB programming environment.

### 4.1 Ten bar truss structure

**Fig. 1:**
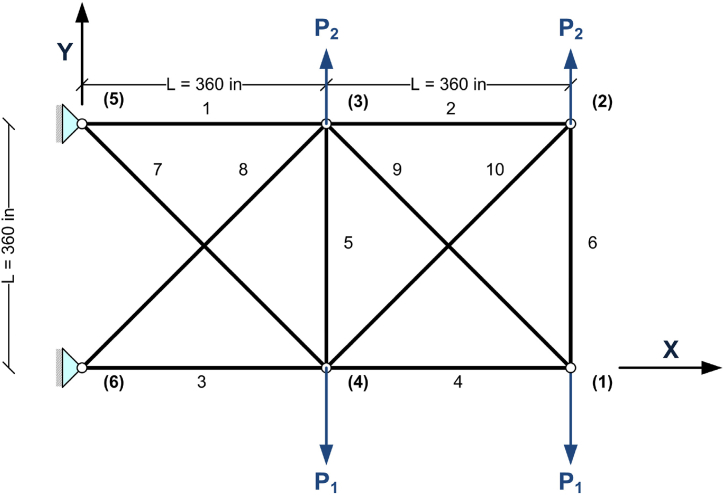
Diagram of the 10 bar truss optimization problem.

The 10 bar truss design optimization problem is a benchmark truss structure that is solved using various methods by many researchers in the past years. The shape of the problem and loadings of it are shown in Fig. 1.

Two loading cases were investigated for this truss structure using the proposed CBSO algorithm in this paper and the quality of the obtained results for this problem are compared with some of the other studies conducted with different methods by other researchers. In the loading case 1, the value for load P_1_ is 100 kips and for load P_2_ there is no load applied and therefore P_2_ is equal to zero. The loading case 2 has values of 150 kips and 50 kips for P_1_ and P_2_ loads, respectively. Mass density for all structural members of this truss design problem is equal to 0.1 lb/in^3^. The elastic modulus is equal to 10000 ksi and each member in this structure is considered as an independent decision variable ranging from 0.1 in^2^ to 35 in^2^. Allowable stress in all members are set to ±25ksi. The allowable nodal displacements for all of the free nodes are set to ±2in. .

### 4.2 Seventeen bar truss structure

**Fig. 2:**
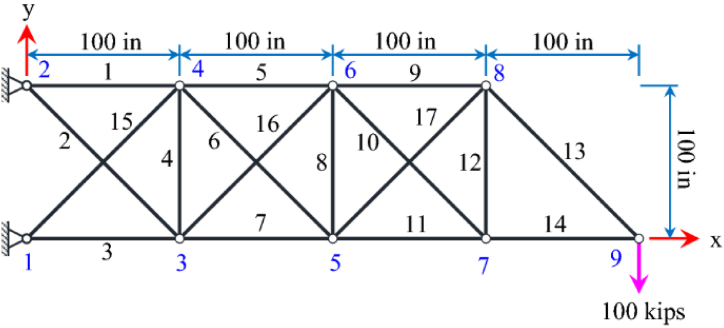
Diagram of the 17 bar truss optimization problem.

The 17-bar truss structure shape and its loading are shown in Fig. 2. In this truss structure, a single vertical load with a value of 100 kips is applied only to node 9. Seventeen design variables are considered for this truss structure and the minimum and the maximum cross section sizes of members for this truss are 0.1 in^2^ and 50 in^2^, respectively. The density of the material used for all members is equal to 0.268 lb/in^3^, the modules of elasticity is 30000 ksi, the stress limit for all members is limited to ±50ksi and the displacement of all nodes in both X and Y directions is set to ±2in. .

### 4.3 Eighteen bar truss structure

**Fig. 3:**
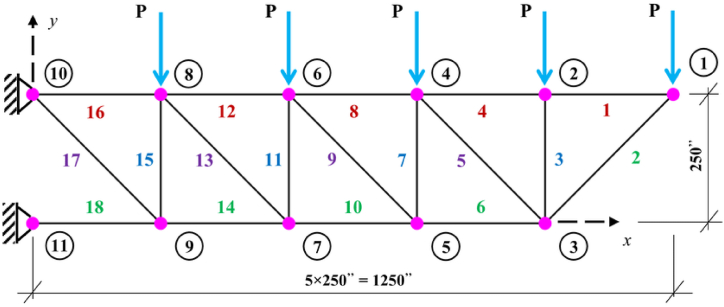
Diagram of the 18 bar truss optimization problem (P = 20 kips).

The third benchmark truss structure investigated in this paper is the 18-bar cantilever truss which is shown in Fig. 3. This structure is subjected to a series of vertical loads in the downward direction, with point loads of 20 kips applied to the upper free nodes. Elastic modulus for all members is 10000 ksi, mass density of all members is 0.1 lb/in^3^ and the stress limit for members in both tension and compression is set to 20 ksi in both X and Y directions. This truss structure optimum design problem, has buckling constraints for its compressive members which are calculated using the following formula:

(14) $$\sigma_{i}^{b}=-\frac{KEA_{i}}{L_{i}^{2}}$$

where A_i_, L_i_, E, and K are cross sectional area, the length of the compressive load bearing members, modulus of elasticity of members and the effective length factor, respectively. The effective length factor value depends on the geometry of the cross section and is assumed to be 4, in this structure. The decision variables for this structure are divided into four groups and the members are linked as follows:

A_1_ = 1,4,8,12,16; A_2_ = 2,6,10,14,18; A_3_ = 3,7,11,15; A_4_ = 5,9,13,17;

The minimum and maximum size of cross sections are 0.1 in^2^ and 50 in^2^ respectively. There are no nodal displacement limitations for the 18-bar truss structure. There are 18 tension and 18 buckling constraints, which makes the total number of nonlinear design constraints 36.

### 4.4 Twenty-five bar truss structure

**Fig. 4:**
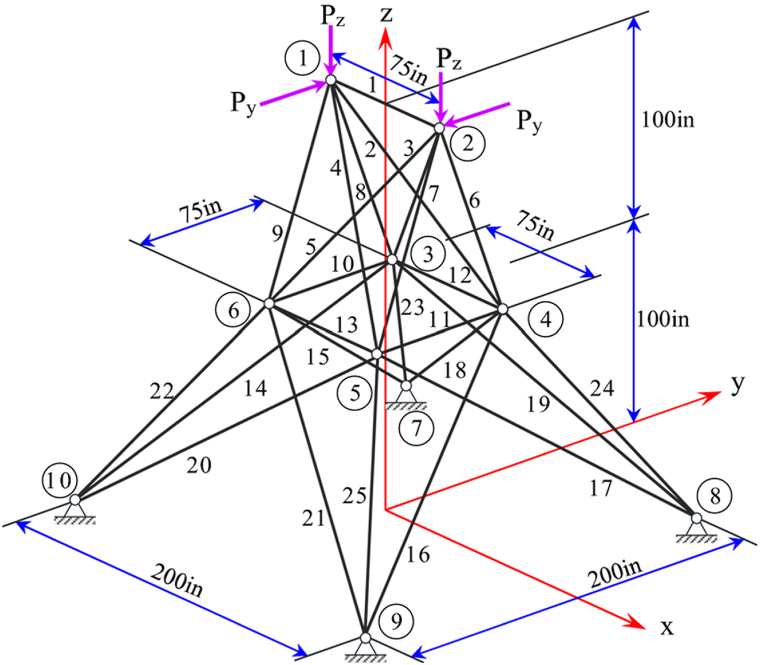
Diagram of the 25 bar truss optimization problem (at node 1:P_Y_ = 20 kips and P_Z_ = −5 kips, at node 2: P_Y_ = −20 kips and P_Z_ = −5 kips).

The geometry of the 25-bar truss structure is depicted in Fig. 4. The mass density value for all materials used in this benchmark truss structure is 0.1 lb/in^3^ and the elastic modulus is 10000 ksi. This truss is symmetric about both X and Y axes and the members of it are grouped into 8 design variables. The members are also subjected to stress limitations as it is shown in Table 1. The minimum and maximum allowable values for cross-sectional members of this truss structure are set to 0.01 in^2^ and 3.4 in^2^, respectively. The free nodes of the 25-bar truss are allowed to displace ±0.35in, in the X, Y and Z directions. Loading conditions for this optimization problem are shown in Table 2. There are 124 nonlinear design constraints considered for this problem.

**Table 1:** Stress limitations(ksi) for members of the 25-bar truss structure [62].

Design variable	Member Grouping	Tensile limit	Compressive limit
1	A1	40	35.092
2	A2∼A5	40	11.590
3	A6∼A9	40	17.305
4	A10∼A11	40	35.092
5	A12∼A13	40	35.092
6	A14∼A17	40	6.759
7	A18∼A21	40	6.759
8	A22∼A25	40	11.082

**Table 2:** Loading conditions (kips) for the 25-bar truss structure [62].

Node	X	Y	Z
1	0	20	−5
2	0	−20	−5
3	0	0	0
6	0	0	0

### 4.5 Seventy-two bar truss structure

**Fig. 5:**
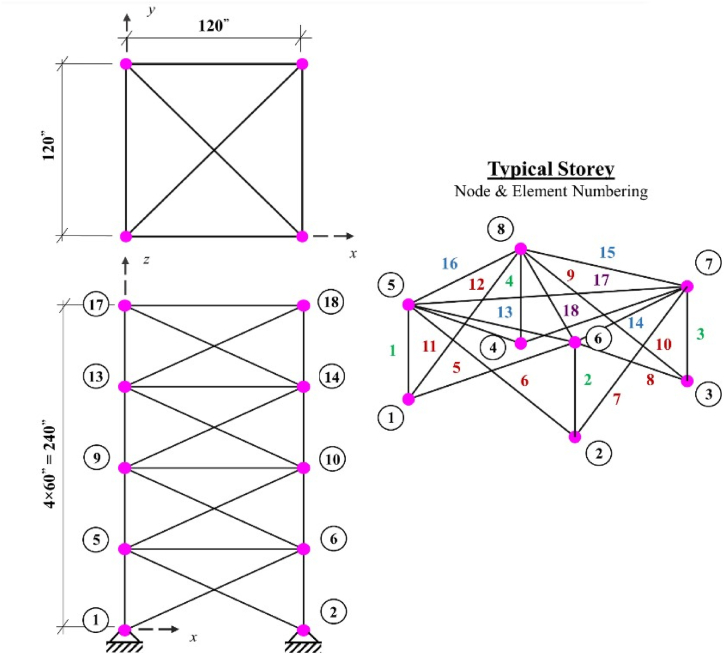
Diagram of the 72 bar truss optimization problem.

The schematic representation of the 72-bar truss tower is presented in Fig. 5. The members of the 72-bar truss optimization problem, are grouped into 16 decision variables and all have the mass density of 0.1 lb/in^3^. The elastic modulus of the used material in this truss is 10000 ksi. The members of the 72-bar truss are subjected to the 25 ksi stress limitation in both tension and compression. The nodal displacement for nodes 17,18,19 and 20 is limited to ±0.25in in the X and Y directions. There are two independent loading conditions considered for this truss structure which are shown in Table 3. This optimization problem is comprised of 320 nonlinear design constraints. The minimum value for cross sections in case 1 is set to 0.1 in^2^ and for case 2 it is equal to 0.01 in^2^. The maximum value of the cross sections in both cases is 4 in^2^.

**Table 3:** Loading conditions (kips) for the 72-bar truss structure [62].

Node	Case 1 X	Case 1 Y	Case 1 Z	Case 2 X	Case 2 Y	Case 2 Z
17	5	5	−5	0	0	−5
18	0	0	0	0	0	−5
19	0	0	0	0	0	−5
20	0	0	0	0	0	−5

### 4.6 One-hundred and 20 bar truss structure

**Fig. 6:**
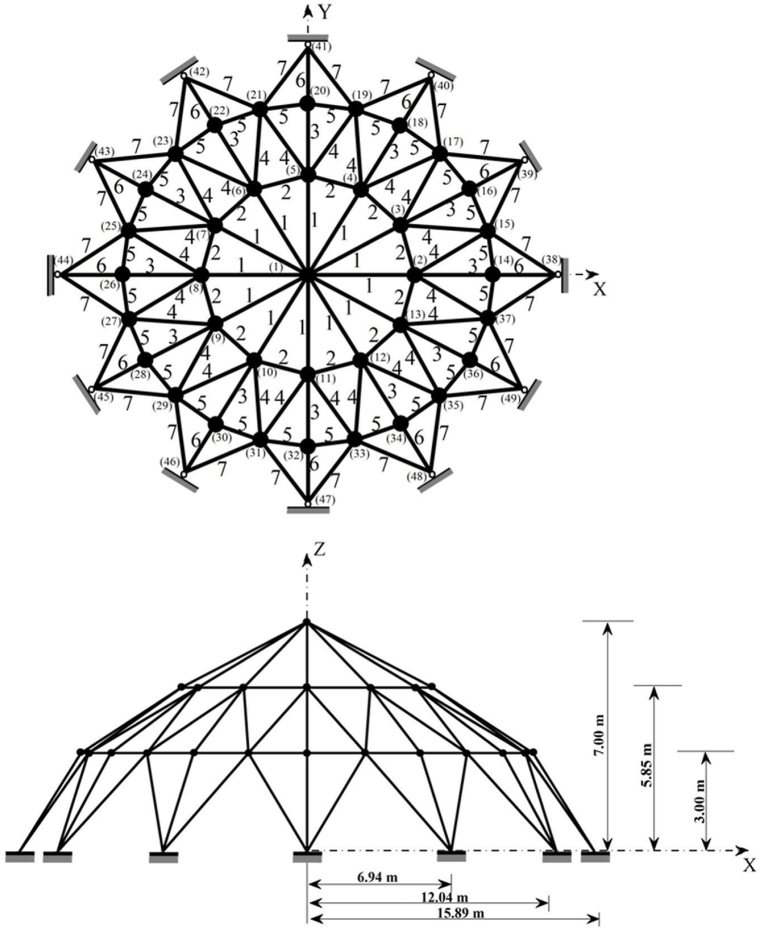
Diagram of the 120 bar truss optimization problem.

The last truss optimization problem considered in this paper is the 120-bar truss structure, and its geometrical representation is depicted in Fig. 6.

The material density for all members of this truss is 0.288 lb/in^3^, the elastic modulus is 30450 ksi, the maximum allowable displacement for all free nodes in all directions is ±0.1969in, and the stress constraints imposed on this truss can be mathematically shown as follows:

(15) $$\sigma_{i}^{\max}=0.6F_{y}\;if\;\sigma_{i}\geq 0$$

(16) $$\sigma_{i}^{\min}\;if\;\sigma_{i}<0$$

Where $F_{y}$ can be set to 58 ksi, which is the yield stress of steel and the value of $\sigma_{i}^{\min}$ can be calculated using equation (17).

(17) $$\sigma_{i}^{\min}=\left\{\begin{array}{c} \frac{\left[\left(1-\frac{\lambda_{i}^{2}}{{2C}_{c}^{2}}\right)F_{y}\right]}{\frac{5}{3}+\frac{3\lambda_{i}}{C_{c}}-\frac{\lambda_{i}^{3}}{{8C}_{c}^{3}}}\;if\lambda_{i}<C_{c}\\ \frac{12\pi^{3}E}{23\lambda_{i}^{2}}\;if\;\lambda_{i}\geq C_{c} \end{array}\right\}$$

Where *E* is the elastic modulus, $C_{c}=\sqrt{2\pi^{2}E/F_{y}}$, $\lambda_{i}=kL_{i}/r_{i}$ is the slenderness ratio and the value of k is set to 1 that denotes the effective length factor, $L_{i}$ is the length of the element *i*, $r_{i}$ shows the radius of gyration and its value can be obtained through $r_{i}=\alpha A_{i}^{\beta }$ with α=0.4993 and β=0.6777 and $A_{i}$ is the cross-sectional area of the element *i*.

**Fig. 7:**
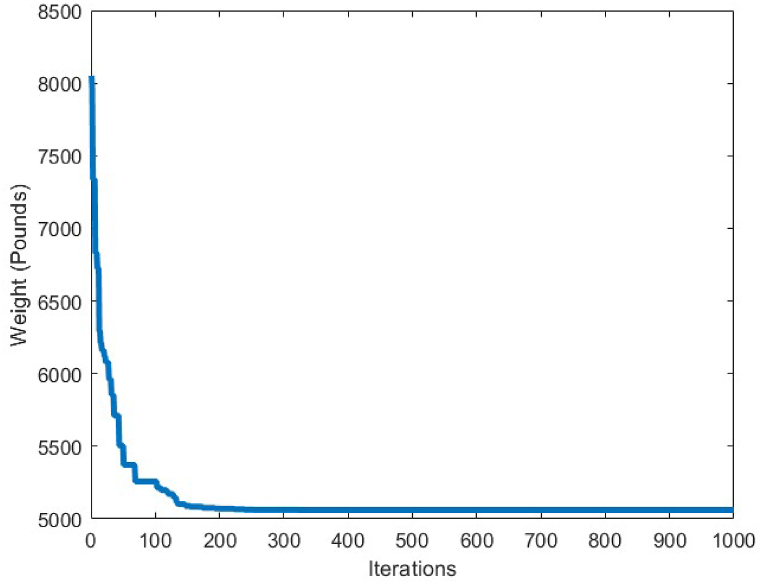
Convergence plot of 10-bar truss case 1.

The applied loads to this truss structure are vertical loads of −13.49 kips at node 1, -6.744 kips at nodes 2 to 14, and -2.248 kips at the other free nodes. The 120 members constructing this truss optimization problem, are connected to each other as shown in Fig. 6, Fig. 7 decision variables are considered to carry-out the optimization process on this structure. The minimum and maximum limitation of the cross-sections for this truss, are set to 0.775 in^2^ and 20 in^2^, respectively.

## 5 Results and discussion

This section presents the performance of the proposed method in the optimum design of the numerical examples discussed in the previous section and the obtained results are compared with some of the available studies in the literature. The presented results by the CBSO algorithm were better than or close to the other compared methods almost in all of the investigated cases. Therefore, it can be stated that the presented method can be considered as a robust and powerful metaheuristic algorithm for solving truss optimum design structures. Based on the obtained results, the CBSO algorithm can be very effective in solving truss sizing optimum design problems and its results are quite acceptable. The obtained results for optimum design of truss structures utilizing the CBSO can be considered as very acceptable achievements in terms of fast convergences and robust optimum designs that highlights the great performance of the presented method in dealing with truss sizing optimum design problems.

### 5.1 Results of 10 bar truss structure case 1

Table 4 shows a comparison of the obtained results utilizing the CBSO algorithm and the other metaheuristic algorithms used to solve the 10-bar truss optimum design problem (case 1 of the 10-bar truss). The CBSO can be ranked as the first algorithm in terms of providing Best weight solution for this optimum design problem, since it can present a better optimum solution for the 10-bar case 1 truss optimum design problem when compared to the other methods. The Best weight reported for the case 1 of this truss structure by the CBSO algorithm is 5060.85 lb, with a total number of 20000 structural evaluations. This optimum weight is the same as the optimum weight obtained in the study of Awad [62], wherein a value of 5060.85 lb was achieved as an optimum design weight. Therefore, the optimum design results obtained by other methods except these two methods were heavier than the optimum design solution presented using the CBSO method. Algorithmic stability of the CBSO in dealing with the 10-bar case 1 truss optimization problem can be considered acceptable since the mean value of its optimum design, is very close to the best weight obtained by the CBSO algorithm and the standard deviation value is a small number. Solving this truss optimization problem with the presented method indicates that utilizing CBSO as an optimizer for its structural weight minimization is quite reasonable and can prove the strong ability of balancing exploration and exploitation capability of the CBSO algorithm when conducting search process in the search space of the 10-bar case 1 optimum design problem. It can be perceived that the presented algorithm effectively examined the search space of this optimum design problem. As a result, a successful investigation of the search space was done using the CBSO algorithm which led to a robust optimum design solution.

**Table 4:** optimum results for the 10-bar truss problem (Case 1).

Design variable (in^2^)	Li et al. [63] HPSO	Sonmez [64] (ABC-AP)	Degertekin [65] (EHS)	Degertekin [65] (SAHS)	Degertekin [66] (TLBO)	Kooshkbaghi & Kaveh [67] (ACCS)	Awad [62] (PO)	Present study (CBSO)
A_1_	30.704	30.548	30.208	30.394	30.4286	30.646	30.501	30.520
A_2_	0.100	0.100	0.100	0.100	0.1000	0.1	0.1	0.100
A_3_	23.167	23.180	22.698	23.098	23.2436	23.103	23.198	23.196
A_4_	15.183	15.218	15.275	15.491	15.3677	15.063	15.247	15.224
A_5_	0.100	0.100	0.100	0.100	0.1000	0.1	0.1	0.100
A_6_	0.551	0.551	0.529	0.529	0.5751	0.573	0.55511	0.550
A_7_	7.460	7.463	7.558	7.488	7.4404	7.478	7.4562	7.457
A_8_	20.978	21.058	21.559	21.189	20.9665	21.094	21.035	21.037
A_9_	21.508	21.501	21.491	21.342	21.5330	21.532	21.526	21.530
A_10_	0.100	0.100	0.100	0.100	0.1000	0.1	0.1	0.100
Best Weight(lb)	5060.92	5060.880	5062.3 9	5061.42	5060.96	5061.03	**5060.85**	**5060.85**
Mean(lb)	N/A	N/A	5063.73	5061.95	5062.08	5061.07	5061.23	5063.09
Stdev(lb)	N/A	N/A	1.98	0.71	0.79	0.09	0.53	5.5307
Number of analysis	125000	500000	9791	7081	16872	12000	7920	20000

Fig. 7 depicts the convergence curve of the 10-bar truss optimum design problem. It can be understood from the convergence curve, that after 200 iterations of the CBSO algorithm, a convergence towards an optimum solution occurs. Hence, the convergence speed of the proposed method towards the optimum solution is quite fast and the CBSO algorithm does not get stuck in local optima.

**Fig. 8:**
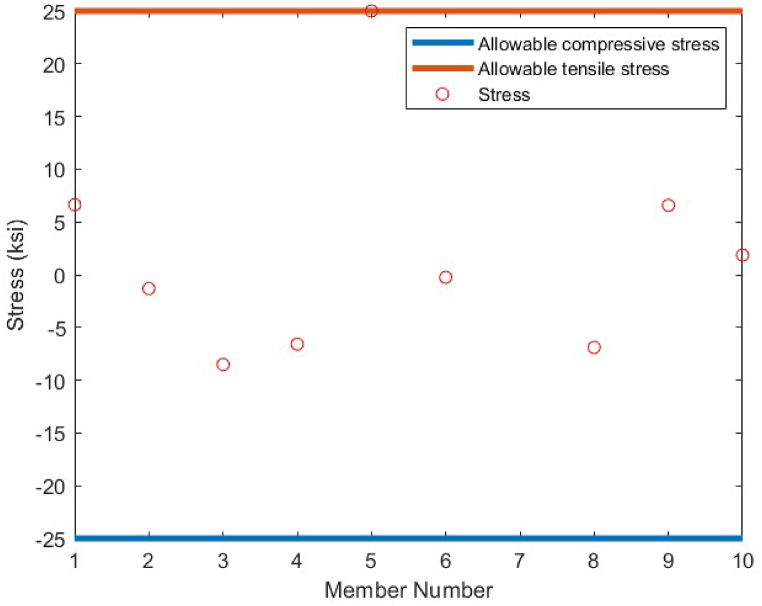
Stress constraint values for 10-bar truss structure case 1.

**Fig. 9:**
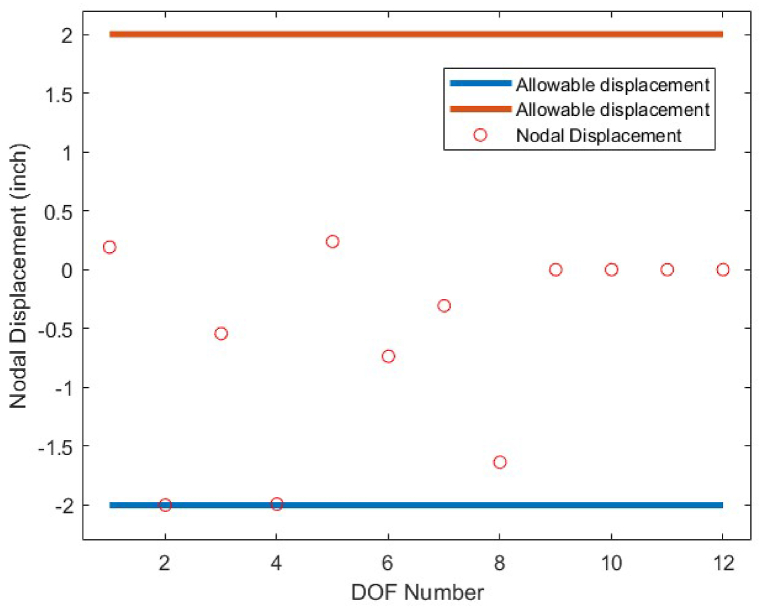
Displacement constraint values for 10-bar truss structure case 1 (DOF = Degree of freedom).

As it is shown in Fig. 8, except member 5 which has a tensile stress of 25 ksi, the other members have stress values between −25 ksi and 25 ksi. There is no violation of stress constraints for the optimized 10-bar truss case 1 using the CBSO algorithm. Fig. 9 depicts the nodal displacement values of the degrees of freedom for the optimized 10-bar truss structure case 1.

### 5.2 Results of 10 bar truss structure case 2

**Fig. 10:**
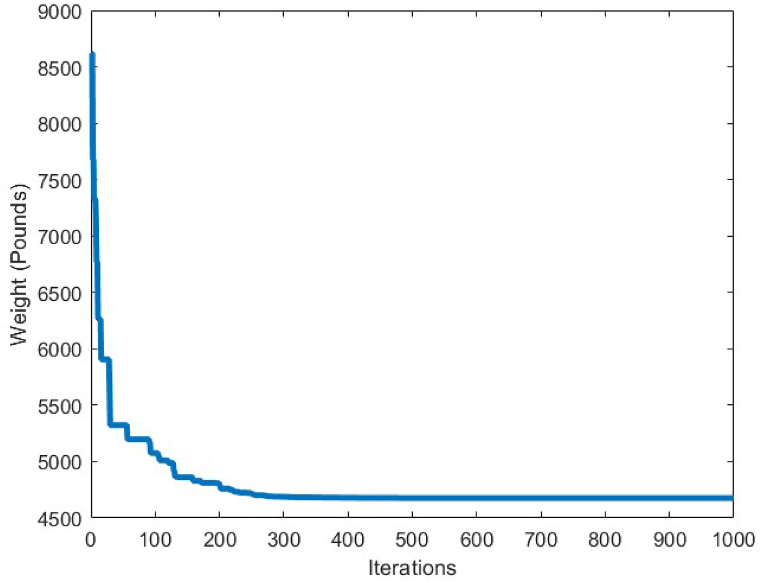
Convergence plot of 10-bar truss case 2.

**Fig. 11:**
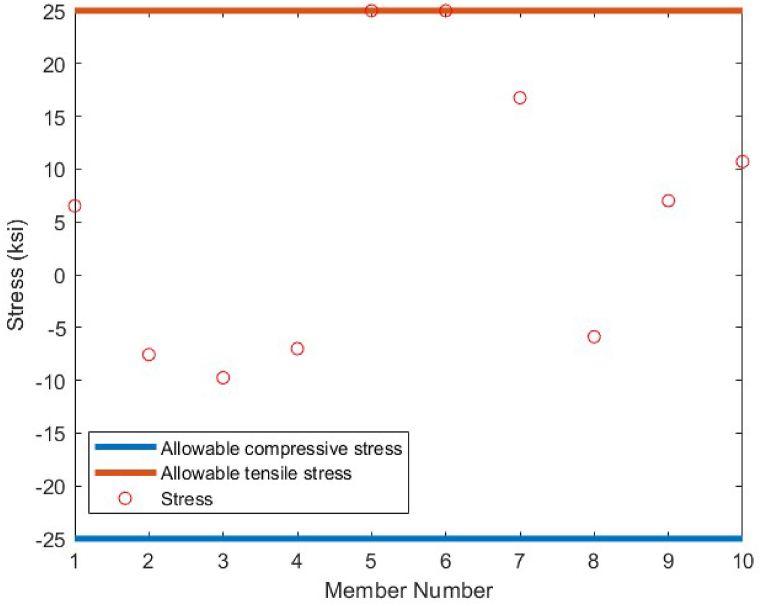
Stress constraint values for 10-bar truss structure case 2.

**Fig. 12:**
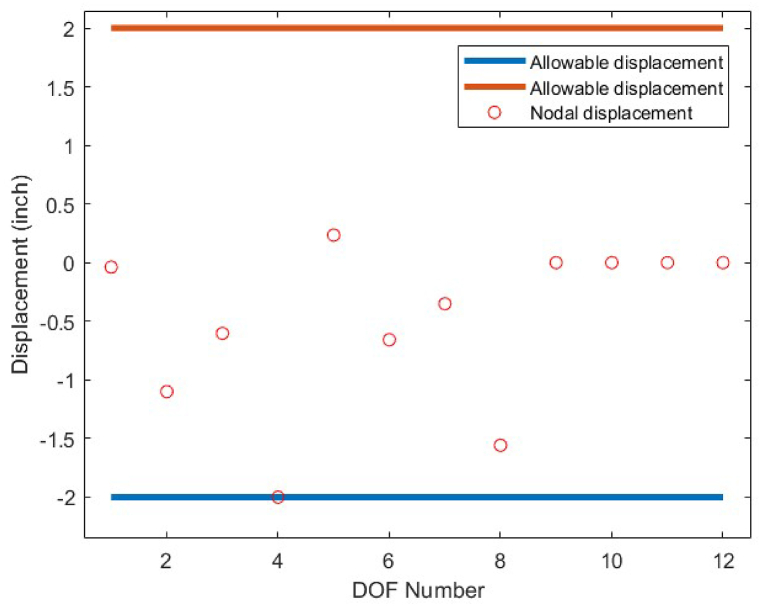
Displacement constraint values for 10-bar truss structure case 2(DOF = Degree of freedom).

Table 5 shows the optimum design results obtained for the second loading case of the 10 bar truss structure. According to the obtained results, the proposed method successfully outperforms the other methods used to compare the achieved results utilizing the CBSO algorithm, in terms of the best weight, the mean weight and the standard deviation values. The closest rival for the CBSO in this case of optimum design, is the optimum design investigated in the study of Kaveh & Zolghadr, in which the best weight obtained was 4677.16 lb. The CBSO presented a promising minimum weight design for this truss structure and can be considered as an efficient method for providing an optimum design solution for the second loading case of the 10 bar truss structure. Fig. 10 shows the convergence curve of the second loading case of the 10-bar truss optimum design problem. Fig. 11, Fig. 12 show the stress constraints values and displacement constraints values for the second loading case of the 10-bar truss structure, respectively.

**Table 5:** Optimum results for the 10-bar truss problem (Case 2).

Design variable (in^2^)	Kaveh & Bakhshpoori [68] (WEO)	Degertekin [66] (TLBO)	Kaveh & Zolghadr [69] (CPA)	Kaveh et al. [70] (PGO)	Li et al. [63] (HPSO)	Awad [62] (PO)	Present study (CBSO)
A_1_	23.5804	23.524	23.5515	23.5326	23.353	23.62	23.534
A_2_	0.1003	0.1000	0.1000	0.1000	0.100	0.1	0.100
A_3_	25.1582	25.441	25.5440	25.0068	25.502	25.434	25.287
A_4_	14.1801	14.479	14.1674	14.4241	14.250	14.351	14.378
A_5_	0.1002	0.1000	0.1000	0.1000	0.100	0.10003	0.100
A_6_	1.9708	1.995	1.9698	1.9721	1.972	1.9701	1.969
A_7_	12.4511	12.334	12.3533	12.4286	12.363	12.339	12.386
A_8_	12.9349	12.689	12.8167	12.8215	12.984	12.712	12.815
A_9_	20.3595	20.354	20.3302	20.4603	20.356	20.346	20.337
A_10_	0.1001	0.1000	0.1001	0.1000	0.1010	0.1	0.100
Best Weight(lb)	4677.31	4678.31	4677.16	4677.17	4677.29	4677.06	**4676.92**
Mean(lb)	4679.06	4680.12	4678.62	4677.88	N/A	4677.97	4677.211
Stdev(lb)	2.07	1.016	0.95	0.72	N/A	0.33	0.3357
Number of analysis	19890	14857	23640	17580	125000	7920	20000

In summary, the CBSO algorithm was quite efficient in both of the two load cases defined for the 10-bar truss optimum design problem compared to the other methods used to optimize this structure.

### 5.3 Results of 17 bar truss structure

**Fig. 13:**
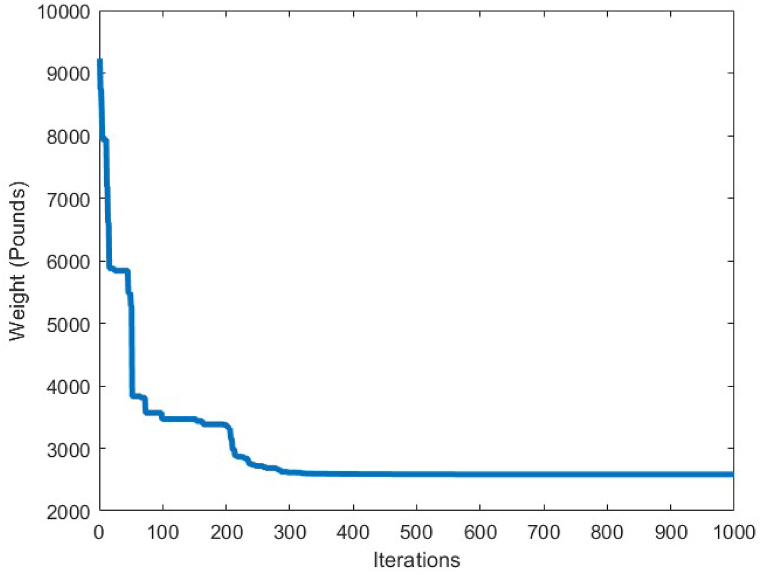
Convergence plot of 17-bar truss.

Table 6 shows the statistical results obtained for optimum design of the 17-bar truss structure utilizing the CBSO and the compared algorithms. Among the results presented in Table 6, the CBSO produced the lightest weight for the analyzed truss structure which is 2581.93 lb, after conducting 50000 structural analysis evaluations. Although the number of structural analysis evaluations by the CBSO algorithm is greater than the other compared methods, however the CBSO successfully outperformed the other methods in terms of providing a better optimum design weight and optimum solution for the considered optimization problem. The stability of the CBSO algorithm in dealing with this optimization problem can be considered quite acceptable as the mean weight for 30 independent runs, is obtained a very close value to that of the best weight achieved by the CBSO algorithm and the standard deviation value is also a relatively small number. According to Fig. 13, CBSO has a good capability in converging towards the optimum solution as it can be observed form the convergence curve that is converged almost at iteration 300. Therefore, despite the great value for the number of structural analysis of this optimization problem stipulated in Table 6, the CBSO algorithm shows very promising behavior in fast convergence and finding an optimum design solution for the 17-bar truss structure problem. In this regard, the optimum solution provided by the CBSO can be an indication of a better optimum design, compared to the other methods presented in Table 6.

**Table 6:** Optimum results for the 17-bar truss problem.

Design variable (in^2^)	Koohestani & Kazemzadeh Azad [71] (ARCGA)	Hadidi et al. [72] (ABC)	Hadidi et al. [72] (MABC)	Hasançebi et al. [73] (SOPT)	Present study (CBSO)
A_1_	15.891	12.9587	15.6762	15.7803	15.9175
A_2_	0.105	0.1	0.1	0.1	0.1041
A_3_	12.101	11.5965	12.0491	12.0897	12.0716
A_4_	0.1	0.1	0.1	0.1	0.1
A_5_	8.075	6.3320	8.1312	8.0825	8.0524
A_6_	5.541	6.5356	5.62020	5.6171	5.5937
A_7_	11.97	12.4792	11.8822	11.9724	11.8799
A_8_	0.1	0.1	0.1	0.1004	0.1
A_9_	7.955	9.0901	8.0517	7.9277	7.9060
A_10_	0.1	0.1	0.1	0.1013	0.1000
A_11_	4.07	5.1578	4.0912	4.0259	4.0831
A_12_	0.1	0.1	0.1	0.1023	0.1001
A_13_	5.705	6.4197	5.6746	5.6875	5.6759
A_14_	3.975	4.0553	3.9864	3.9905	3.9891
A_15_	5.516	5.7984	5.6729	5.5159	5.5592
A_16_	0.1	0.1	0.1	0.1	0.1009
A_17_	5.563	6.8470	5.4907	5.6292	5.5927
Weight(lb)	2581.95	2642.45	2582.27	2582.09	**2581.93**
Mean(lb)	N/A	N/A	N/A	N/A	2582.12
Stdev(lb)	N/A	N/A	N/A	N/A	0.2201
Number of analysis	10000	20000	20000	6617	50000

**Fig. 14:**
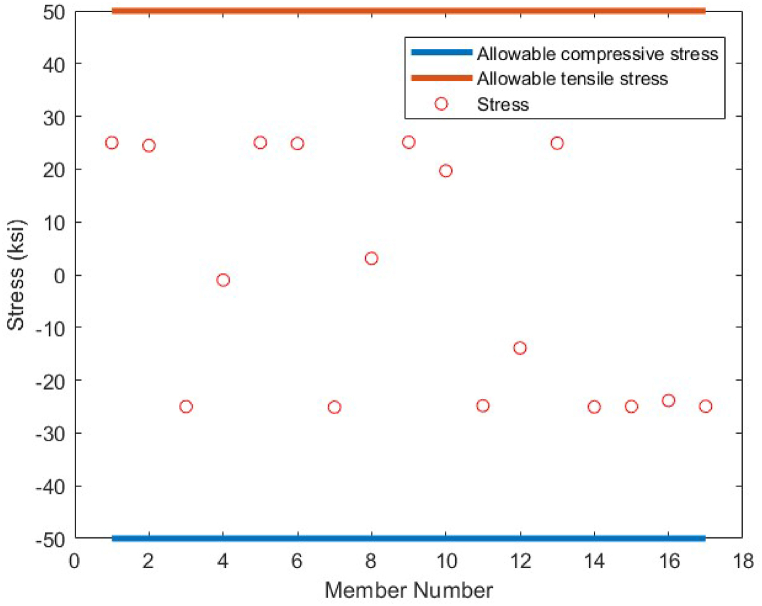
Stress constraint values for 17-bar truss structure.

**Fig. 15:**
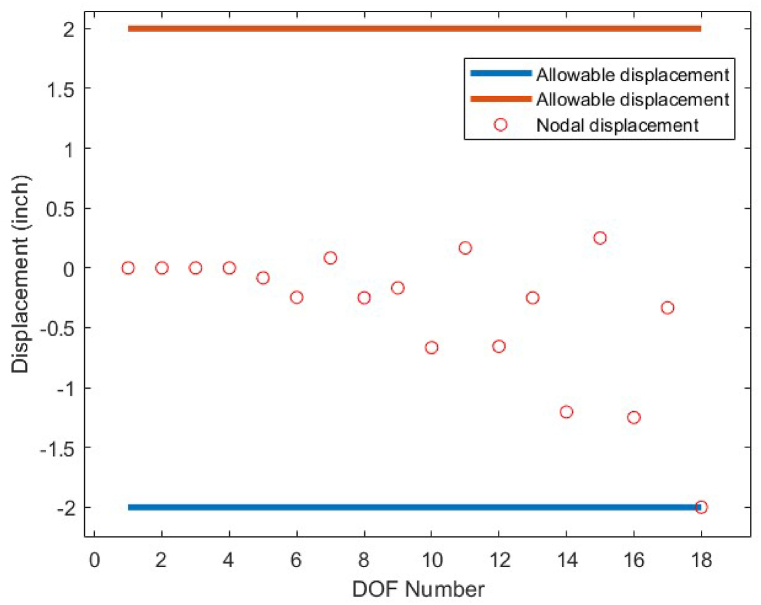
Displacement constraint values for 17-bar truss structure (DOF = Degree of freedom).

Fig. 13 shows the convergence curve of the 17-bar truss structure obtained utilizing the CBSO algorithm. Fig. 14, Fig. 15 show the stress and displacement constraints values for the 17-bar truss structure, respectively.

### 5.4 Results of 18 bar truss structure

**Fig. 16:**
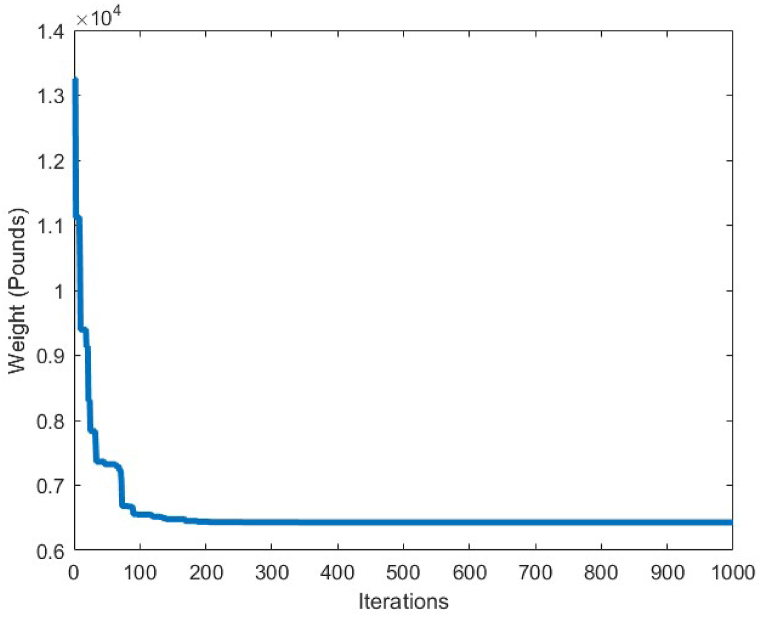
Convergence plot of 18-bar truss.

**Fig. 17:**
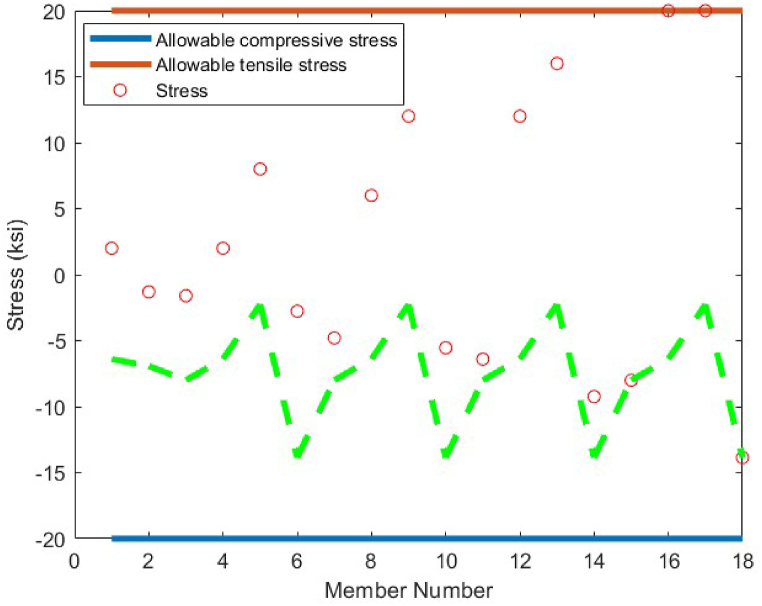
Stress constraint values for 18-bar truss structure (Green dashed line shows the buckling limits).

The statistical results obtained by the CBSO algorithm and the other methods that studied the 18-bar truss optimum design problem are presented in Table 7. The CBSO algorithm provides acceptable optimum design solution when examined with and compared to the other methods in Table 7. The number of structural analysis conducted by the CBSO, in order to find an optimum solution for the 18-bar truss structure is 10000 which is less than that of studies carried out by Khatibinia & Yazdani [76], and Sonmez [64]. There were slight violations of the defined constraints for this truss structure in the studies of Lee & Geem [75] and Imai & Schmit [74], in which the optimum design of this truss structure was assessed and investigated. Overall, the performance of the CBSO algorithm in dealing with this optimum design problem was quite acceptable, since the obtained results are quite close to that of the other available studies presented in Table 7. Fig. 16 shows the convergence curve for the optimum design of the 18-bar truss structure and it is obvious that near the iteration 200, the presented algorithm, is almost converged towards the optimum solution that is considered a fast convergence towards the optimum solution. There are no constraint violations for the optimum design of the 18-bar truss structure provided by the CBSO algorithm, which do exist in the studies of Lee & Geem [75] and Imai & Schmit [74] as mentioned earlier. This can also be observed in Fig. 17, where the stresses in members are within the defined limits of the imposed constraints of this optimization problem.

**Table 7:** Optimum results for the 18-bar truss problem.

Design variable (in^2^)	Imai & Schmit [74] (Multiplier Method)	Lee & Geem [75] (HS)	Sonmez [64] (ABC-AP)	Khatibinia & Yazdani [76] (MGSA)	Khatibinia & Yazdani [76] (AMGSA)	Awad [62] (PO)	Present study (CBSO)
A_1_	9.998	9.980	10.000	10.000	10.000	10.000	10.000
A_2_	21.65	21.63	21.651	21.651	21.651	21.651	21.650
A_3_	12.50	12.49	12.500	12.500	12.500	12.500	12.500
A_4_	7.072	7.057	7.071	7.071	7.071	7.071	7.071
Weight(lb)	6430	6421.88	6430.529	6430.529	6430.529	6430.529	6430.529
Mean(lb)	N/A	N/A	N/A	6431	6430.529	6430.529	6430.529
Stdev(lb)	N/A	N/A	N/A	0.0	0.0	0.0	5.45E-04
Number of analysis	N/A	2000	200000	15000	10500	3564	10000

### 5.5 Results of 25 bar truss structure

**Fig. 18:**
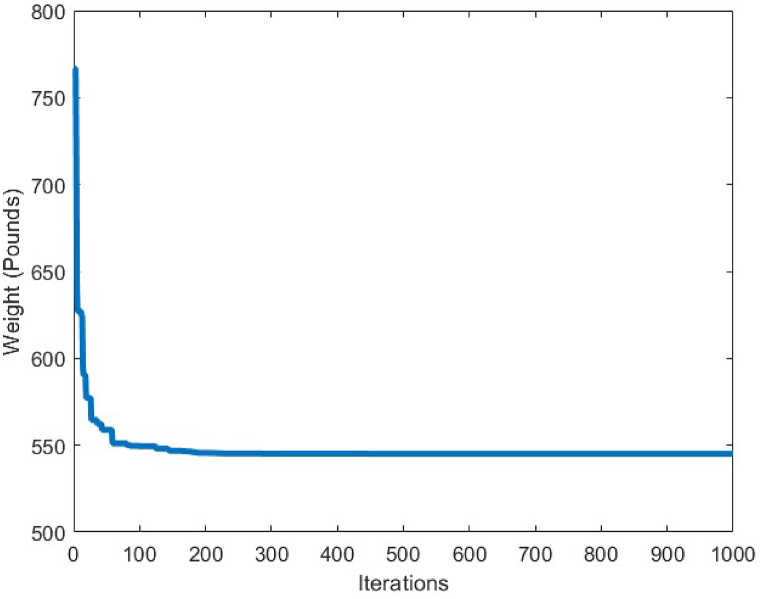
Convergence plot of 25-bar truss.

**Fig. 19:**
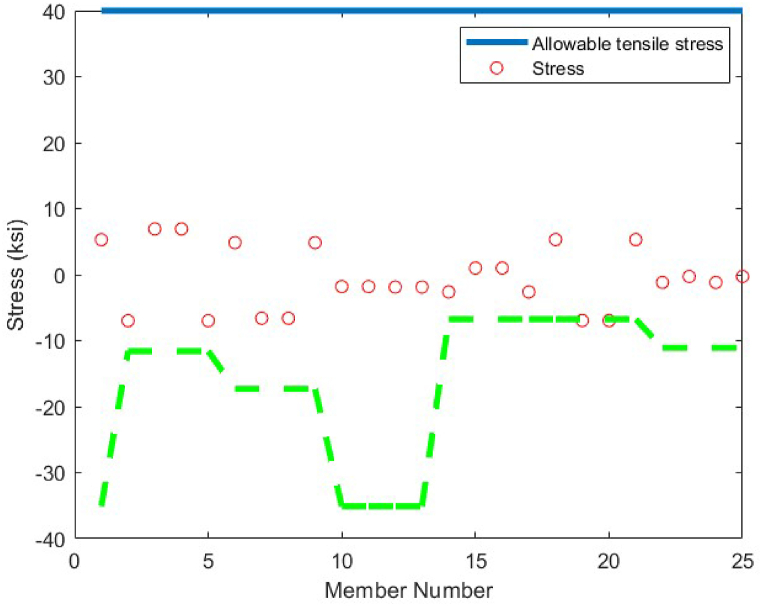
Stress constraint values for 25-bar truss structure (Green dashed line shows the allowable compressive stress).

**Fig. 20:**
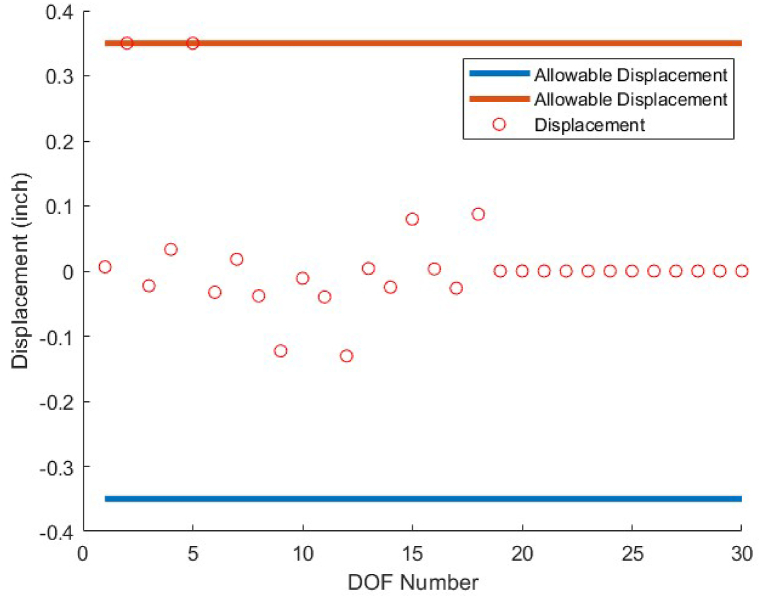
Displacement constraint values for 25-bar truss structure (DOF = Degree of freedom).

A comparison between the results of different algorithms that were utilized to solve the 25-bar truss structure problem and the CBSO algorithm, is presented in Table 8. The CBSO algorithm successfully outperforms the other methods in terms of the best weight, mean weight and the standard deviation values. The closest rival of the CBSO in dealing with this optimization problem is the HPSSO algorithm that was utilized by Kaveh et al., to solve this optimum design problem. The CBSO algorithm also shows a very good convergence speed towards the optimum solution, since it is almost converged to an optimum solution after 200 iterations as it is depicted in Fig. 18. Therefore, the presented CBSO, can be considered as an efficient optimization algorithm and an effective optimizer in solving the 25-bar truss optimum design problem, as it provides an improvement in the light weight design of this structure over the other methods used to solve this problem. Fig. 19, Fig. 20, depict the stress and displacement constraint values for the optimum design of the 25-bar truss structure, respectively.

**Table 8:** Optimum results for the 25-bar truss problem.

Design variable (in^2^)	Camp & Bichon [76] (ACO)	Li et al. [63] (PSO)	Li et al. [63] (PSOPC)	Kaveh & Zakiyan [78] (BA)	Kaveh et al. [79] (HPSSO)	Kaveh & Zolghadr [69] (CPA)	Awad [62] (PO)	Present study (CBSO)
A_1_	0.0100	9.863	0.010	0.0100	0.01	0.0100	0.010038	0.0100
A_2_	2.0000	1.798	1.979	1.97889	1.9907	1.9890	1.9797	1.9870
A_3_	2.9660	3.654	3.011	3.00472	2.9881	2.9880	3.0052	2.9934
A_4_	0.0100	0.100	0.100	0.01000	0.0100	0.0100	0.01	0.0100
A_5_	0.0120	0.100	0.100	0.01000	0.0100	0.0100	0.010018	0.0100
A_6_	0.6890	0.596	0.657	0.68880	0.6824	0.6980	0.68165	0.6839
A_7_	1.6790	1.659	1.678	1.67834	1.6764	1.6780	1.6779	1.6768
A_8_	2.6680	2.612	2.693	2.65270	2.6656	2.6580	2.6617	2.6621
Weight(lb)	545.530	627.08	545.27	545.168	545.164	545.18	545.165	**545.162**
Mean(lb)	546.340	N/A	N/A	546.446	545.556	545.49	545.45	545.1627
Stdev(lb)	0.94	N/A	N/A	N/A	0.432	0.24	0.21	1.74E-06
Number of analysis	16500	150000	150000	20000	13326	22800	8316	20000

### 5.6 Results of 72 bar truss structure case 1 and case 2

**Fig. 21:**
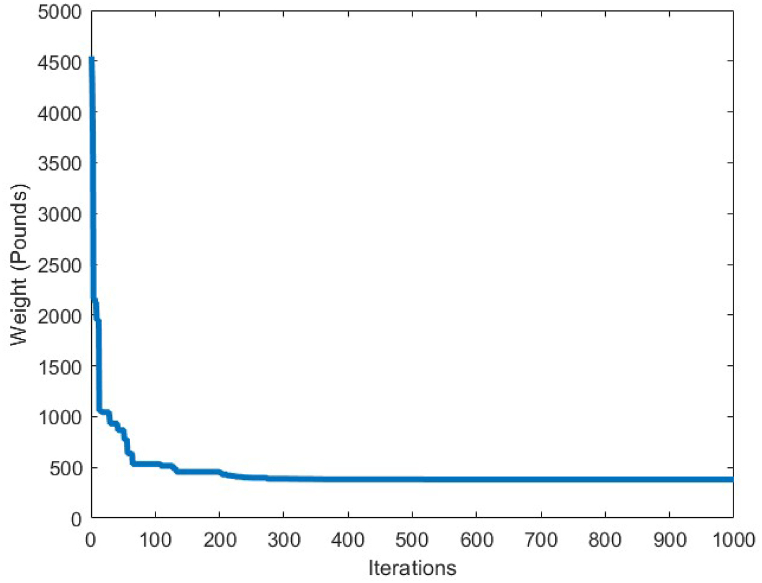
Convergence plot of 72-bar truss(Case 1).

**Fig. 22:**
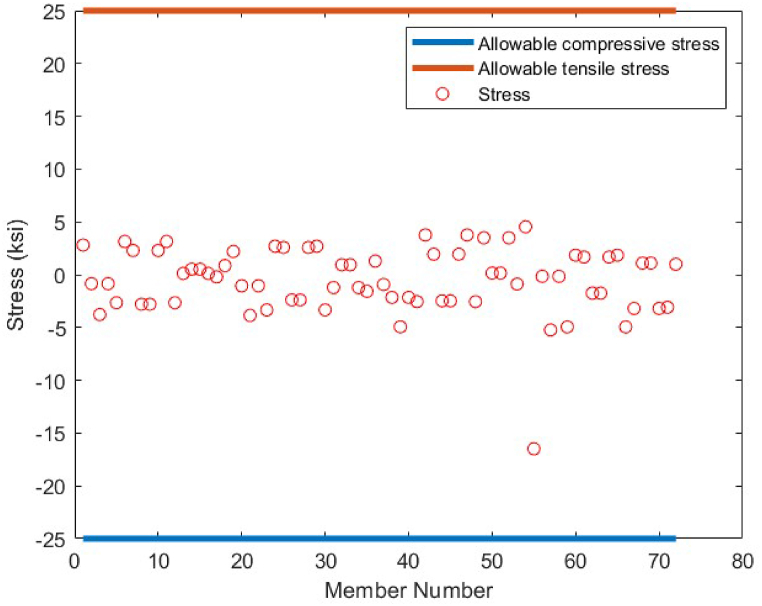
Stress constraint values for 72-bar truss structure(Case 1).

**Fig. 23:**
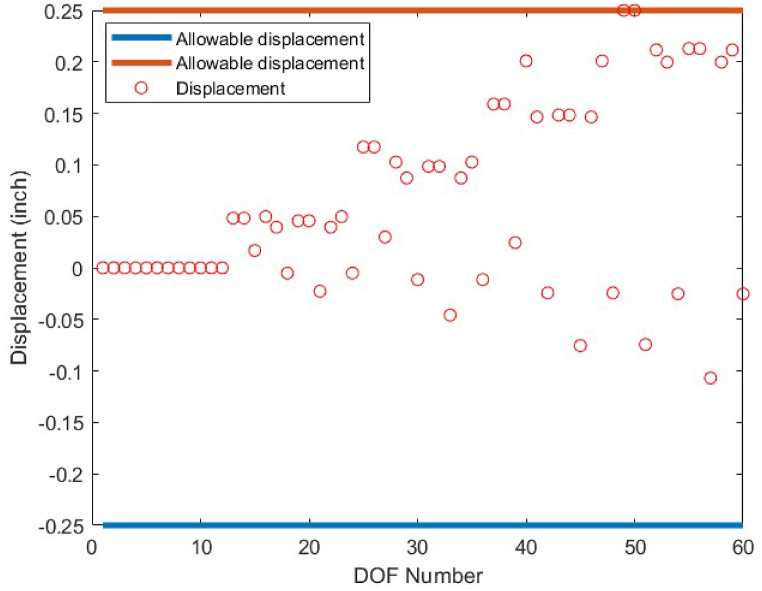
Displacement constraint values for 72-bar truss structure case 1 (DOF = Degree of freedom).

**Fig. 24:**
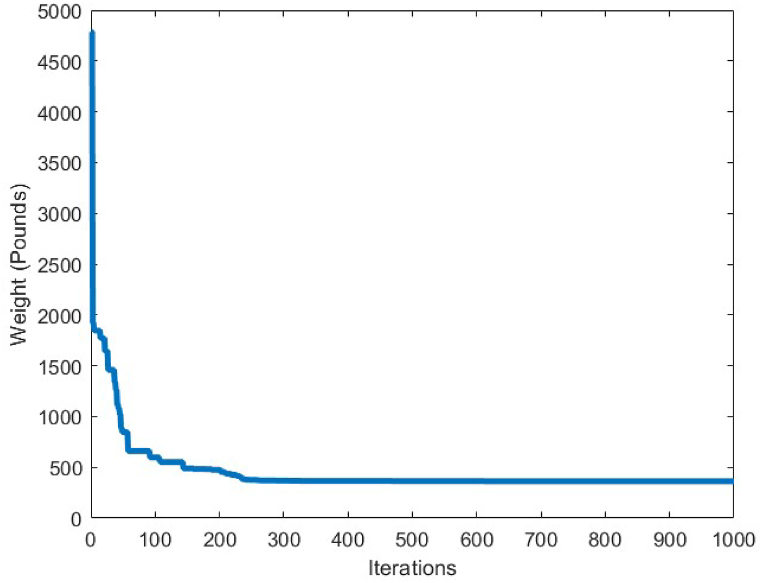
Convergence plot of 72-bar truss(Case 2).

**Fig. 25:**
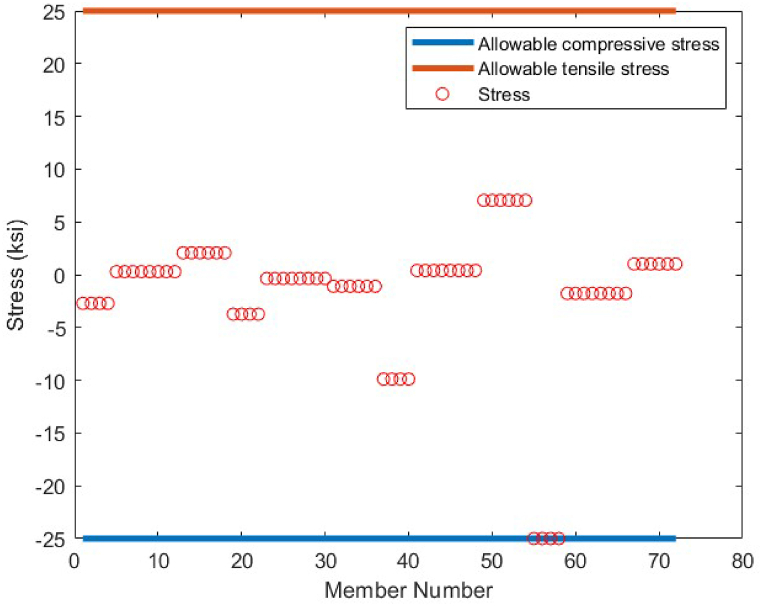
Stress constraint values for 72-bar truss structure(Case 2).

**Fig. 26:**
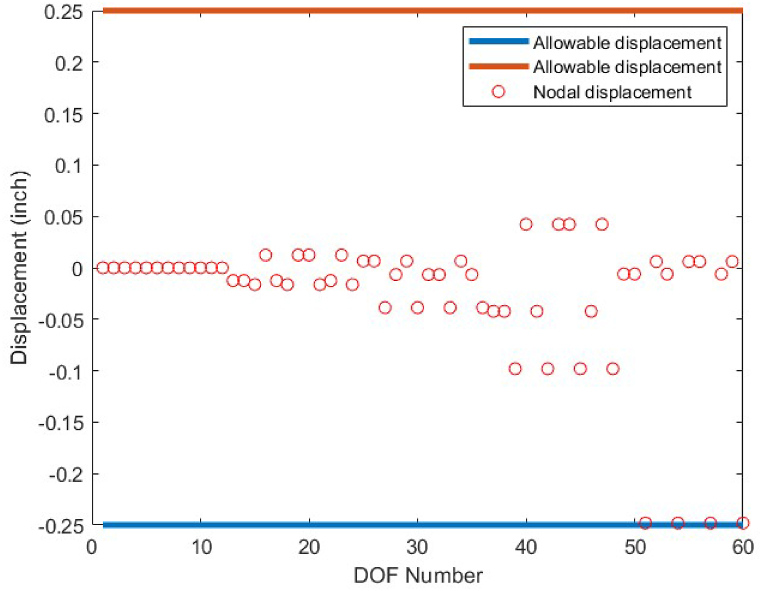
Displacement constraint values for 72-bar truss structure case 2 (DOF = Degree of freedom).

In order to conduct an analysis on the obtained results by the CBSO algorithm in optimum design of the 72-bar truss structure, and also investigate its performance in dealing with this optimization problem, Table 9 is presented that indicates a clear advantage of the CBSO in solving this problem in terms of being a superior metaheuristic over the other methods utilized in the literature, since it provides a better optimum solution with a lighter weight structure. Although the CBSO algorithm could not outperform all of the other existing methods in the second case of the 72-bar truss structure, but the optimum design weight obtained by the presented CBSO algorithm is very close to that of the study carried out by Degertekin. Therefore, the optimum design by the CBSO can be considered acceptable. It is also worthwhile noting that the CBSO ranks first in the second case of optimum design of the 72-bar truss structure when we analyze the mean weight and the standard deviation of the statistical analysis results provided in Table 10. As it can be seen from Fig. 21, the CBSO has a fast convergence rate towards the optimum solution in dealing with the 72-bar truss structure optimum design problem. Almost near the iteration 300, it is converged, which can be deemed as an advantage of the presented method. The fast convergence to the optimum design solution can be noticed in the case 2 of the 72-bar truss problem, in which the CBSO algorithm was able to find the optimum solution in the early iterations of the presented algorithm. The defined stress and displacement constraints were checked and the obtained stresses in members and the nodal displacements by the CBSO optimum design are shown for both cases. Fig. 22, Fig. 23, show the stress and displacement constraints values for the first loading case of the 72-bar truss structure, respectively. Fig. 24, presents the convergence curve of the second loading case of the optimum design of the 72-bar truss structure. A fast convergence can be seen from the convergence curve and according to Fig. 25, Fig. 26, there are no constraint violations for the second case of the 72-bar truss structure.

**Table 9:** Optimum results for the 72-bar truss problem(Case 1).

Design variable (in^2^)	Camp et al. [77] (ACO)	Kaveh & Khayatazad [80] (RO)	Kaveh & Zakiyan [78] (BA)	Malihe et al. [81] (CA)	Ozbasaran & Yildirim [82] (CSA)	Awad [62] (PO)	Present study (CBSO)
A_1_	1.948	1.8365	1.85920	1.7892	1.5995	1.8607	1.8635
A_2_	0.508	0.5021	0.49308	0.4571	0.6009	0.50626	0.5130
A_3_	0.101	0.1000	0.10025	0.1002	0.1005	0.1	0.1000
A_4_	0.102	0.1004	0.10178	0.1000	0.1105	0.1	0.1002
A_5_	1.303	1.2522	1.28534	1.3200	1.2956	1.2749	1.2681
A_6_	0.511	0.5033	0.51307	0.5427	0.4685	0.50952	0.5081
A_7_	0.101	0.1002	0.10073	0.1000	0.10001	0.1	0.1000
A_8_	0.100	0.1002	0.10248	0.1000	0.2368	0.10012	0.1000
A_9_	0.561	0.5730	0.51214	0.5380	0.3954	0.52872	0.5219
A_10_	0.492	0.5499	0.52547	0.5377	0.5015	0.52359	0.5207
A_11_	0.1	0.1004	0.10029	0.1000	0.1008	0.10014	0.1000
A_12_	0.107	0.1001	0.10297	0.1000	0.1035	0.10001	0.1001
A_13_	0.156	0.1576	0.15597	0.1562	0.1805	0.15628	0.1564
A_14_	0.550	0.5222	0.55473	0.5593	0.6675	0.54998	0.5473
A_15_	0.390	0.4356	0.40627	0.4360	0.36	0.40132	0.4094
A_16_	0.592	0.5972	0.59617	0.5619	0.7107	0.58582	0.5807
Weight(lb)	380.24	380.458	380.05819	380.925	394.7116	379.68	**379.6585**
Mean(lb)	383.16	382.5538	389.14389	382.374	426.4132	379.79	379.7445
Stdev(lb)	3.66	N/A	N/A	0.67	24.07941	0.139	0.0684
Number of analysis	18500	19084	20000	19650	150000	7788	50000

**Table 10:** Optimum results for the 72-bar truss problem(Case 2).

Design variable (in^2^)	Lee & Geem [75] (HS)	Li et al. [63] (PSOPC)	Sonmez [64] (ABC-AP)	Degertekin [66] (TLBO)	Kaveh et al. [68] (WEO)	Awad [62] (PO)	Present study (CBSO)
A_1_	1.963	1.652	1.8907	1.8929	1.8616	1.8812	1.8899
A_2_	0.481	0.547	0.5166	0.5160	0.5206	0.52207	0.5142
A_3_	0.010	0.100	0.0100	0.0100	0.0105	0.01003	0.0100
A_4_	0.011	0.101	0.0100	0.0100	0.0100	0.010024	0.0100
A_5_	1.233	1.102	1.2968	1.2917	1.2455	1.3045	1.2922
A_6_	0.506	0.589	0.5191	0.5176	0.5177	0.51551	0.5175
A_7_	0.011	0.011	0.0100	0.0100	0.0101	0.010011	0.0100
A_8_	0.012	0.010	0.0101	0.0100	0.0100	0.010067	0.0101
A_9_	0.538	0.581	0.5208	0.5229	0.5327	0.52429	0.5243
A_10_	0.533	0.458	0.5178	0.5193	0.5109	0.51519	0.5185
A_11_	0.010	0.010	0.0100	0.0100	0.0100	0.011122	0.0101
A_12_	0.167	0.152	0.1048	0.0997	0.1205	0.10444	0.1201
A_13_	0.161	0.161	0.1675	0.1680	0.1655	0.16738	0.1657
A_14_	0.542	0.555	0.5346	0.5359	0.5397	0.53737	0.5386
A_15_	0.478	0.514	0.4443	0.4457	0.4554	0.44244	0.4455
A_16_	0.551	0.648	0.5803	0.5818	0.5995	0.57549	0.5628
Weight(lb)	364.33	368.45	363.8683	**363.841**	363.9827	363.88	363.8548
Mean(lb)	N/A	N/A	N/A	364.42	364.3536	364.315	364.0248
Stdev(lb)	N/A	N/A	N/A	0.49	0.2188	0.181	0.2037
Number of analysis	20000	125000	400000	17954	19860	8448	50000

### 5.7 Results of one hundred and 20 bar truss structure

**Fig. 27:**
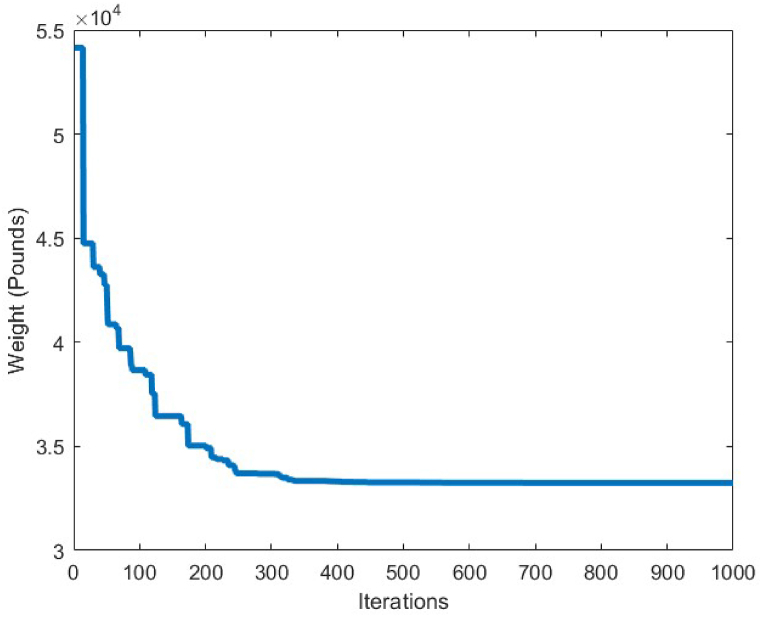
Convergence plot of 120-bar truss.

**Fig. 28:**
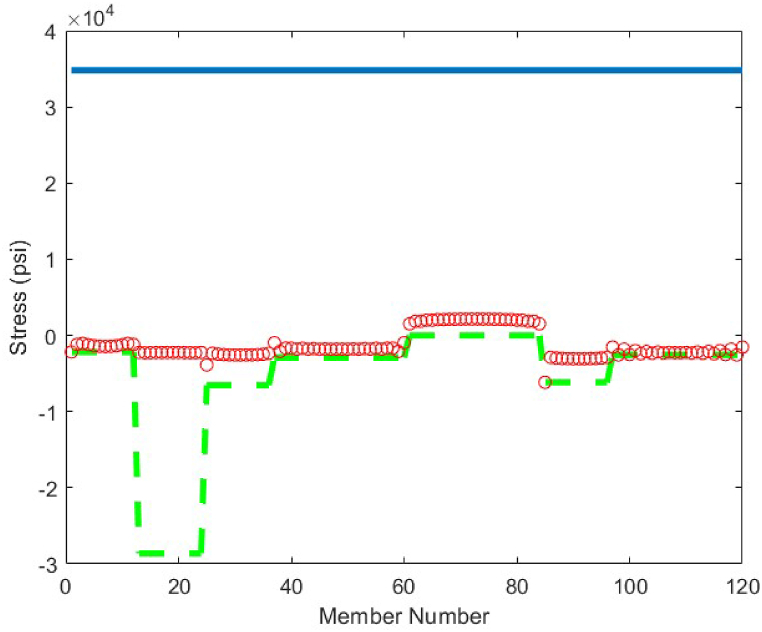
Stress constraint values for 120-bar truss structure (Blue lines shows allowable tensile stress, dashed green line shows the allowable compressive stress and red circles show member stress).

**Fig. 29:**
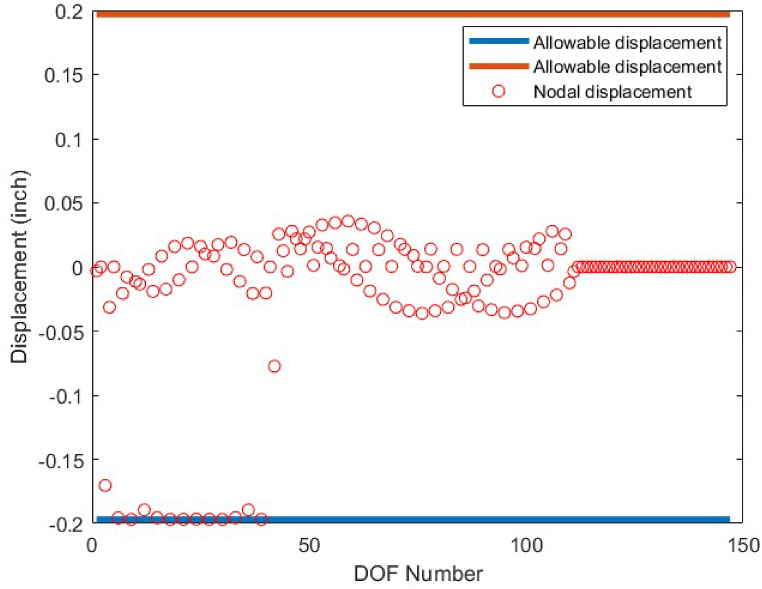
Displacement constraint values for 120-bar truss structure (DOF = Degree of freedom).

The last optimum design truss structure investigated in this paper is the 120-bar truss. Table 11 shows that the CBSO algorithm outperforms the other methods in terms of the best weight, mean weight and the standard deviation values. Almost after 320 iterations, the CBSO converged to the optimum solution which describes a relatively fast convergence towards the optimum solution of the 120-bar truss structure. According to Fig. 28, that presents the stress values in the optimum design provided by the CBSO, a large number of the 120-bar truss structure's members have a really close compressive stress values to the defined compressive stress constraints of this optimization problem that shows the presented optimum design can be considered economic and safe. In Fig. 29, the nodal displacements of the degrees of freedom of the problem are presented with the related limitations for displacements. Fast convergence of the presented method in dealing with the 120-bar truss optimum design problem can be seen in Fig. 27.

**Table 11:** Optimum results for the 120-bar truss problem.

Design variable (in^2^)	Li et al. [63] (PSO)	Li et al. [63] (PSOPC)	Kaveh & Khayatazad [83] (RO)	Kooshkbaghi & Kaveh [67] (ACCS)	Present study (CBSO)
A_1_	12.802	3.040	3.030	3.0243	3.0243
A_2_	11.765	13.149	14.806	14.7924	14.7884
A_3_	5.654	5.646	5.440	5.0594	5.0835
A_4_	6.333	3.143	3.124	3.1361	3.1364
A_5_	6.963	8.759	8.021	8.4828	8.4721
A_6_	6.492	3.758	3.614	3.2984	3.2842
A_7_	4.988	2.502	2.487	2.4967	2.4964
Weight(lb)	51986.2	33481.2	33317.8	33250.2846	**33249.5351**
Mean(lb)	N/A	N/A	N/A	33255.2668	33249.7715
Stdev(lb)	N/A	N/A	N/A	3.1126	0.1511
Number of analysis	N/A	N/A	N/A	5700	50000

## 6 Time complexity

In order to evaluate performance of the presented method in dealing with the truss optimum design problems in this paper, an investigation on the required run time for solving the analyzed optimum design problems using the CBSO is conducted in this section. The mean run time for presenting and finding an optimum solution for each of the examined problems is presented here to have an understanding of the amount of time that takes to find optimum solutions of the analyzed truss design problems by the CBSO.

**Table:** 

Optimization problem	Mean of run-time for 30 runs(seconds)
Ten-bar truss case 1	3.74 s
Ten-bar truss case 2	3.71 s
Seventeen-bar truss	13.22 s
Eighteen-bar truss	3.26 s
Twenty-five bar truss	8.47 s
Seventy-two bar truss (Case 1)	43.58 s
Seventy-two bar truss (Case 2)	43.52 s
One-hundred and 20 bar truss	82.19 s

## 7 Matlab code for CBSO

clear all; clc;

n_PoP = 150;

M_Iter = 1000;

n_dim = 10; lb = [−100 -100 -100 -100 -100 -100 -100 -100 -100 -100]; ub = [100 100 100 100 100 100 100 100 100 100]; Obj_Func = @(x)cec17_func(x',1); Best_X = zeros(1,n_dim); Best_fit = inf; Conv_Curve = zeros(1,M_Iter); fit_val = inf(n_PoP,1); X = initialization(n_PoP,n_dim,ub,lb); XL = repmat(ones(1,n_dim).∗lb,n_PoP,1); XU = repmat(ones(1,n_dim).∗ub,n_PoP,1); Iter = 0; while Iter < M_Iter

for i = 1:size(X,1)

Flag4ub = X(i,) > ub;

Flag4lb = X(i,) < lb;

X(i,)=(X(i,).∗(∼(Flag4ub + Flag4lb))) + ub.∗Flag4ub + lb.∗Flag4lb; fit_val(i,1) = Obj_Func(X(i,)); if fit_val(i,1) < Best_fit.

Best_fit = fit_val(i,1); Best_X = X(i,); end

end

if Iter = = 0

fit_old = fit_val; X_old = X; end.

Inx=(fit_old < fit_val);

Indx = repmat(Inx,1,n_dim); X = Indx.∗X_old+∼Indx.∗X;

fit_val = Inx.∗fit_old+∼Inx.∗fit_val;

fit_old = fit_val; X_old = X;

E = repmat(Best_X,n_PoP,1); CF = rand∗(1-Iter/M_Iter); LD = levy(n_PoP,n_dim,1.5); ND = randn(n_PoP,n_dim); for i = 1:size(X,1)

SEL1 = randi([1 i],1); SEL2 = randi([1 i],1); for j = 1:size(X,2)

R = rand(); if Iter<0.2∗M_Iter.

X(i,j) = X(i,j)+(ND(i,j)∗(rand∗E(i,j)-X(i,j))); elseif Iter ≥ 0.2∗M_Iter && Iter ≤ 0.4∗M_Iter

if i > size(X,1)/2.

X(i,j) = E(i,j) + CF∗(ND(i,j)∗(rand∗E(i,j)-X(SEL1,j))); else.

X(i,j) = X(i,j)+(LD(i,j)∗(E(i,j)-LD(i,j)∗X(SEL2,j))); end

else.

X(i,j) = E(i,j) + CF∗(LD(i,j)∗(LD(i,j)∗X(SEL1,j)-X(SEL2,j))); end

end

end

for i = 1:size(X,1)

Flag4ub = X(i,) > ub;

Flag4lb = X(i,) < lb;

X(i,)=(X(i,).∗(∼(Flag4ub + Flag4lb))) + ub.∗Flag4ub + lb.∗Flag4lb; fit_val(i,1) = Obj_Func(X(i,)); if fit_val(i,1) < Best_fit.

Best_fit = fit_val(i,1); Best_X = X(i,); end

end

if Iter = = 0

fit_old = fit_val; X_old = X; end.

Inx=(fit_old < fit_val);

Indx = repmat(Inx,1,n_dim); X = Indx.∗X_old+∼Indx.∗X;

fit_val = Inx.∗fit_old+∼Inx.∗fit_val;

fit_old = fit_val; X_old = X; if rand()<0.2.

X = X + rand∗(CF∗XL + rand(n_PoP,n_dim).∗(XU-XL)); else

r = rand(); XS = size(X,1); X = X + rand∗rand∗(X(randperm(XS),)-X(randperm(XS),)); end.

Iter = Iter+1; Conv_Curve(Iter) = Best_fit; disp(['Iter ', num2str(Iter), ': ', num2str(Conv_Curve(Iter))]); end

function Positions = initialization(n_PoP,n_dim,ub,lb)

B_S = size(ub,2); if B_S = = 1.

Positions = rand(n_PoP,n_dim).∗(ub-lb) + lb; end

if B_S > 1

for i = 1:n_dim

ub_i = ub(i); lb_i = lb(i); Positions(:,i) = rand(n_PoP,1).∗(ub_i-lb_i) + lb_i; end

end

end

function [z] = levy(n,m,beta)

num = gamma(1+beta)∗sin(pi∗beta/2); den = gamma((1+beta)/2)∗beta∗2^((beta-1)/2); sigma_u = (num/den)^(1/beta);

u = random('Normal',0,sigma_u,n,m);

v = random('Normal',0,1,n,m);

z = u./(abs(v).^(1/beta)); end.

## 8 Graphical explanation and flowchart of the CBSO

**Fig. 30:**
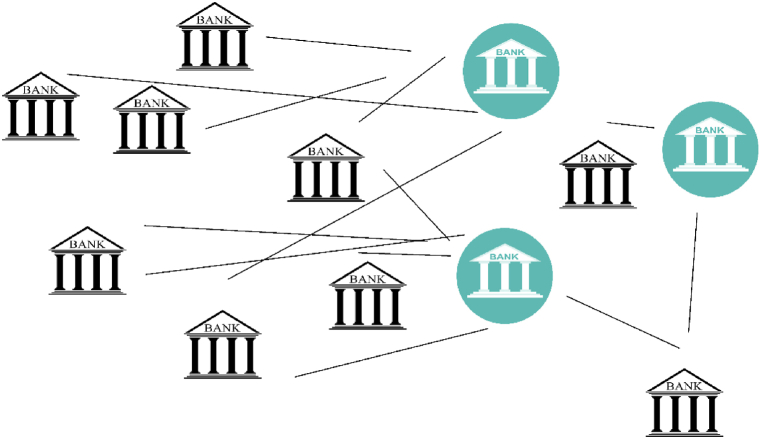
Graphical explanation of Phase 1.

**Fig. 31:**
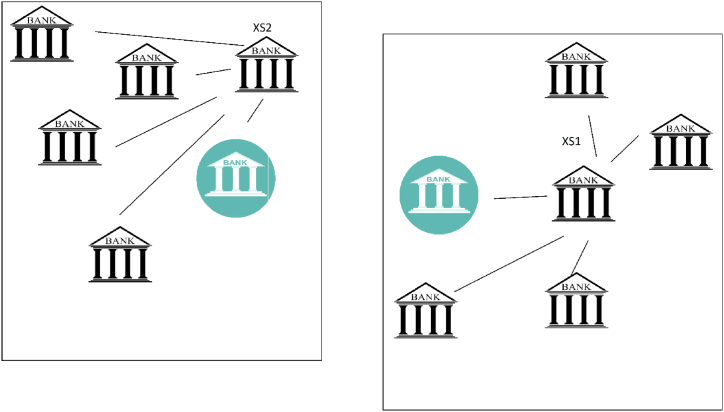
Graphical explanation of Phase 2.

**Fig. 32:**
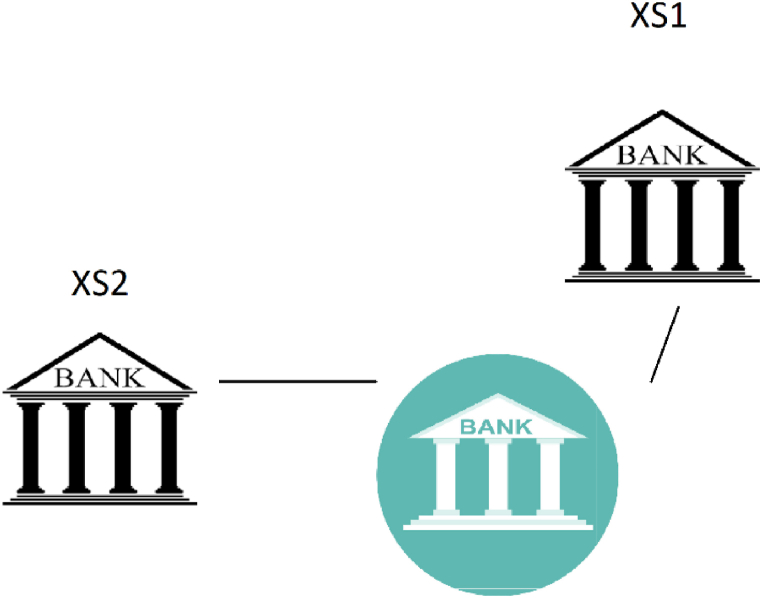
Graphical explanation of Phase 3.

**Fig. 33:**
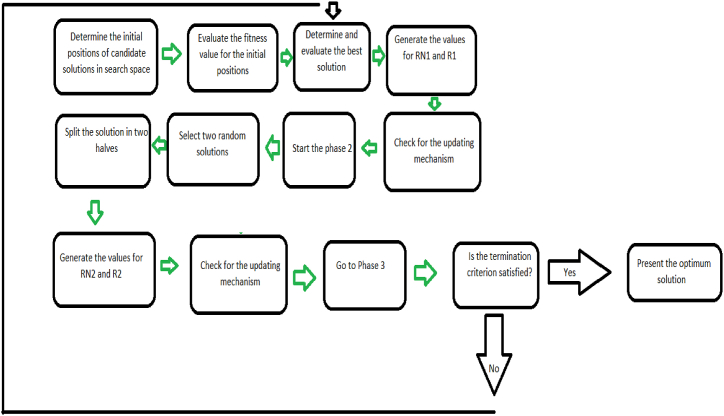
Flowchart of the CBSO.

In order to give a brief graphical abstract and explanation of the CBSO algorithm, Fig. 30, Fig. 31, Fig. 32 are presented which show the different phases of the CBSO algorithm, respectively. Fig. 33 graphically summarizes the procedures of the CBSO algorithm, and depicts a flowchart for it.

## 9 Conclusions

This paper presented a study on the capability and efficiency of a metaheuristic algorithm inspired by connected banking system in solving truss sizing optimum design problems and the feasibility of providing acceptable light weights for the investigated truss structures with continuous decision variables. In order to conduct a reasonable evaluation of truss optimum design problems, a number of standard truss structures were considered as optimum design benchmarks which involve planar and spatial trusses that are subjected to several constraints and loading conditions. The CBSO algorithm was tested against these benchmark structures (from 10-bar truss structure to 120-bar truss structure) and its capability in solving these extremely challenging optimization problems was proved. It can be realized that the presented optimization method is capable of balancing its own exploration and exploitation capabilities and has a robust ability in rapid converging towards optimal and near optimal solutions of the investigated problems. The presented CBSO algorithm can be seen as a method that utilizes a computer based system technology as an inspiration for its design and implementation as a metaheuristic. The CBSO outperformed the other methods which were used to compare the ability of the presented metaheuristic algorithm with them, in almost all of the investigated cases. The optimum solutions provided by the CBSO were better than the best weights obtained among the other compared methods. According to the obtained results by the CBSO and the different methods used to compare the results provided by the CBSO algorithm, the presented method can be regarded as a powerful and robust optimizer for solving continuous sizing truss optimization problems.

For future directions, analyzing shape/size and topology optimization of structural design problems with the CBSO can be suggested. Investigating the discrete sizing optimization problems with the presented method is also recommended. Using the CBSO algorithm for optimum design of truss structures with dynamic constraints for having a valid evaluation of the performance of the CBSO algorithm can be carried out in future studies.

## Declaration of funding

No funding was received for the research conducted in this study.

## CRediT authorship contribution statement

**Mehrdad Nemati:** Writing – review & editing, Writing – original draft, Visualization, Validation, Software, Resources, Methodology, Investigation, Formal analysis, Conceptualization. **Yousef Zandi:** Supervision. **Jamshid Sabouri:** Supervision.

## Declaration of competing interest

The authors declare that they have no known competing financial interests or personal relationships that could have appeared to influence the work reported in this paper.
